# Interaction between calcium and potassium modulates elongation rate in cotton fiber cells

**DOI:** 10.1093/jxb/erx346

**Published:** 2017-10-13

**Authors:** Kai Guo, Lili Tu, Yonghui He, Jinwu Deng, Maojun Wang, Hui Huang, Zhonghua Li, Xianlong Zhang

**Affiliations:** National Key Laboratory of Crop Genetic Improvement, Huazhong Agricultural University, Wuhan, Hubei, China

**Keywords:** Abscisic acid, calcium, fiber elongation, ionome, jasmonic acid, potassium, ROS

## Abstract

Calcium (Ca^2+^) is necessary for fiber cell development in cotton (*Gossypium hirsutum*), both as a cell wall structural component and for environmental signaling responses. It is also known that potassium (K^+^) plays a critical role in cotton fiber cell elongation. However, it is unclear whether Ca^2+^ integrates its activities with K^+^ to regulate fiber elongation. Here, we report the novel discovery that Ca^2+^ deficiency, when integrated with K^+^ signaling, promotes fiber elongation. Using inductively coupled plasma–mass spectrometry (ICP-MS), we determined dynamic profiles of the ionome in ovules and fibers at different developmental stages, and found that a high accumulation of macro-elements, but not Ca^2+^, was associated with longer fibers. Using an *in vitro* ovule culture system, we found that under Ca^2+^-deficient conditions, sufficient K^+^ (52 mM) rapidly induced ovule and fiber browning, while reduced K^+^ (2 or 27 mM) not only suppressed tissue browning but also altered fiber elongation. Reduced K^+^ also enhanced reactive oxygen species scavenging ability and maintained abscisic acid and jasmonic acid levels, which in turn compensated for Ca^2+^ deficiency. Ca^2+^ deficiency combined with reduced K^+^ (0 mM Ca^2+^ and 27 mM K^+^) produced longer fibers in cultured ovules, due to cell wall loosening by phytosulfokine (PSK), expansin (EXP), and xyloglucan endotransglycosylase/hydrolase (XTH), and an increase of the K^+^ content of fiber cells. Using transgenic cotton, we showed that the *CBL-INTERACTING PROTEIN KINASE 6* (*GhCIPK6*) gene mediates the uptake of K^+^ under Ca^2+^-deficient conditions. This study establishes a new link between Ca^2+^, K^+^, and fiber elongation.

## Teaser

Potassium maintains cotton fiber cell elongation under conditions of calcium deficiency, through the *CBL-INTERACTING PROTEIN KINASE 6* (*GhCIPK6*) gene.

## Introduction

With the current increase in environmental stresses and the shortage of arable land, it is a major challenge to establish efficient and sustainable agricultural production (Schenkel, 2010; Gregory and George, 2011). The improvement of crop productivity and product quality to a large extent depend on a sufficient availability of mineral nutrients (Gregory *et al.*, 2013). Fibers are the main harvest product of cotton (*Gossypium hirsutum*) and provide the essential raw material for a significant part of the textile industry (Qin and Zhu, 2011). Recent research suggests that an improvement of mineral nutrient availability might represent a new approach to increase fiber length.

Fiber cells initiate from the outer-layer cells of the ovule, which undergo 20 d of rapid elongation growth and secondary cell wall synthesis, and after a subsequent period of dehydration become a valuable crop. Fiber cells can extend to 15–40 mm, and also represent an excellent model for studying single-cell elongation (Lee *et al.*, 2007; Haigler *et al.*, 2012). The fiber cell elongates in a linear cell-growth mode, showing characteristics both of tip growth and diffuse growth (Qin and Zhu, 2011). Cell wall loosening, osmotic pressure, and the synthesis of structural molecules are three important determinants for rapid fiber cell elongation (Ruan *et al.*, 2001; Wang and Ruan, 2013). Two key proteins, expansin and endo-1,4-beta-glucanase, maintain the looseness of the fiber cell wall during the elongation stage (Ruan *et al.*, 2001, 2003; Ruan, 2014). Primary cell wall extensibility is also partially mediated by xyloglucan endotransglycosylases/hydrolases (XTHs) that cleave and re-attach the xyloglucan polymers that make up the hemicellulose matrix of type I cell walls (Cosgrove, 2005; Lee *et al.*, 2010). Sugars, organic acids, and potassium maintain cell osmotic pressure and drive horizontal and longitudinal elongation. The basic substrate materials for primary cell wall synthesis include components of the cytoskeleton, cell membrane lipids, and pectin, all of which are necessary to maintain cell elongation (Qin and Zhu, 2011). The mineral elements calcium (Ca^2+^) and potassium (K^+^) also play critical roles in fiber elongation.

Ca^2+^ has an indispensable role in plant development, both as a structural component in lipid membranes and cell walls, and also as a secondary messenger responding to environmental signaling (White and Broadley, 2003; Dodd *et al.*, 2010). Carboxyl groups from opposing pectins in plant cell walls where the cellulose microfibrils are cross-linked can be electrostatically co-ordinated by Ca^2+^, conferring rigidity to cell walls. Ca^2+^ also co-ordinates with phosphate groups from phospholipids to maintain the stability of the plasma membrane (Maathuis, 2009). Ca^2+^ deficiency can lead to cracking, tipburn, or rot in horticultural crops (White and Broadley, 2003).

A tip-focused gradient of cytosolic Ca^2+^ is an important determinant of polarity in tip-growing cells such as root hairs and pollen tubes (Konrad *et al.*, 2011; Mangano *et al.*, 2016). A Ca^2+^ gradient is also set up in the apical zone of cotton fiber cells, which determines the oritentation of fiber elongation and helps the formation of secretory vesicles containing materials for fiber cell growth (Qin and Zhu, 2011). The fluorescent dyes DiOC and Fluo-3/AM reveal a high Ca^2+^ gradient near the growing tip in rapidly elongating cotton fiber cells (Taliercio and Boykin, 2007; Huang *et al.*, 2008). During elongation of fiber cells there is an increased Ca^2+^ influx at the cell tip, whereby the flux rate peaks during plasmodesmatal closure from 10 to 15 d post-anthesis (DPA) (Tang *et al.*, 2014). This Ca^2+^ influx activates downstream intracellular receptors to control fiber development, such as GhCDPK1, GhCaM7, or GhCaM7-like, which are preferentially expressed in the elongating fiber (Tang *et al.*, 2014; Cheng *et al.*, 2016; Zhang *et al.*, 2016). The target proteins of the activated intracellular receptors are involved in the production of reactive oxygen species (ROS), which regulates fiber development in a manner dependent on Ca^2+^ concentration (Wang *et al.*, 2011; Tang *et al.*, 2014; Zhang *et al.*, 2016). An optimum increase of ROS induces sucrose transporters (*GhSUT1* and *GhSUT2-A*) and K^+^ transporters (*GhKT1* and *GhKT2*) to promote fiber elongation (Zhang *et al.*, 2016). Therefore, the possibility exists that the Ca^2+^ signal interacts with the K^+^ osmotic pressure component to promote fiber elongation. However, the details of the mechanism are not clear.

K^+^ homeostasis in plant cells also influences metabolism through the transcriptional and post-transcriptional regulation of metabolic enzymes or their activities (Armengaud *et al.*, 2004, 2009; Maathuis, 2009; Wang and Wu, 2013; Demidchik, 2014). As sink cells, elongating fiber cells require abundant potassium to maintain cell turgor pressure, via the K^+^ transporter genes preferentially expressed in elongating fibers (Ruan *et al.*, 2001; Wang and Ruan, 2013). A recent study has suggested that low K^+^ induces premature senescence in cotton and that soil K^+^ deficiency causes defective fiber properties, including reduced fiber length, fiber strength, and lint weight (Yang *et al.*, 2016*a*, 2016*b*). In our previous research, we showed that a high level of endogenous phytosulfokine (PSK-α) in fibers slowed down the efflux of K^+^ and promoted longer fiber cells (Han *et al.*, 2014). How Ca^2+^ regulates K^+^ uptake in fiber cells through interactions with hormones and ROS during fiber elongation remains to be elucidated.

Ionome quantification integrated with RNA-seq provides a way to investigate the relationship between fiber elongation, gene expression, and mineral element composition. In this study, we quantified ion contents in fibers and ovules during fiber development at different stages, using inductively coupled plasma–mass spectrometry (ICP-MS). Using an ovule culture system, we found that Ca^2+^ starvation promotes early fiber elongation in association with an increased K^+^ content. We have also clarified the relationship between Ca^2+^ and K^+^, expression of *PSK*, *EXP* and *XTH*, and K^+^ uptake by *GhCIPK6* during fiber elongation.

## Materials and methods

### Plant materials

Cotton (*Gossypium hirsutum*) plants TM-1, Xuzhou142, the *Xuzhou142 lintless-fuzzless* mutant (*xu142-fl*), YZ1, and *Gossypium barbadense* 3-79 were grown in the fields of Huazhong Agricultural University, Wuhan, Hubei province, China, under standard farming conditions. In the experimental plot, each cotton variety was grown in a subplot that contained 300 plants in 30 rows. The soil was yellow-brown loam (pH 5.65 ± 0.11; organic matter, 12.12 ± 0.90 g kg^−1^; available nitrogen, 91.41 ± 7.56 mg kg^−1^; available phosphorus, 97.06 ± 18.13 mg kg^−1^; available potassium, 391.79 ± 56.95 mg kg^−1^; total nitrogen 0.47 ± 0.16 g kg^−1^; total phosphorus 10.52 ± 1.23 g kg^−1^; total potassium 7.71 ± 0.0 g kg^−1^; all values are means ±SD). N fertilization (240 kg N ha^−1^), P fertilization (120 kg P_2_O_5_ ha^−1^), K fertilization (150 kg K_2_O ha^−1^), and irrigation were performed in accordance with standard agricultural practices. Bolls were tagged at the day of flowering as 0 d post-anthesis (DPA). At the required development stage, bolls were harvested and frozen in liquid nitrogen, and stored at –70 °C until analysis.

### Ovule culture

To identify the effect of each mineral element on fiber development, ovule culture was carried out with two cotton varieties, *Gossypium hirsutum* TM-1 and YZ1. Ovule culture was performed as described previously (Beasley, 1973; Beasley and Ting, 1973; Guo *et al.*, 2016), with details given in Supplementary Fig. S1 at *JXB* online. Flowers were collected on the afternoon of the day of flowering (0.5 DPA). After the flower organs had been peeled off, ovaries were sterilized with 0.1% HgCl_2_ for 15 min, washed with double-distilled water and cut off. Sterile ovules were stripped off and suspended on the ovule culture medium, which was supplemented with 0.5 µM GA_3_ and 5 µM IAA.

The concentration of each element in the ovule culture medium was set according to the BT medium of Beasley and Ting (1973). The concentration of each chemical was as follows: 50 mM KNO_3_, 2 mM KH_2_PO_4_, 3 mM CaCl_2_, 2 mM MgSO_4_, 100 µM H_3_BO_3_, 100 µM MnSO_4_, 30 µM FeSO_4_, 30 µM ZnSO_4_, 5 µM KI, 1 µM Na_2_MoO_4_, 0.1 µM CoCl_2_, 0.1µM CuSO_4_, vitamins (4 µM VB1, 4 µM VB6, and 3.2 µM VB3), 1 mM inositol, and 120 mM glucose, at pH 5.0. Six concentrations were set up for each essential element (K, P, Ca, Mg, Fe, Zn, Mn, B, Mo, Co, and Cu), which were 0, 0.5-, 1-, 2-, 4-, and 8-fold the standard (1-fold) concentration in the medium. For the series concentration for K^+^, KNO_3_ was replaced by NH_4_NO_3_ in medium with an equivalent molarity of nitrogen: K0 indicates that 50 mM KNO_3_ was replaced by 25 mM NH_4_NO_3_, and K0.5 indicates that 50 mM KNO_3_ was replaced by 25 mM KNO_3_ and 12.5 mM NH_4_NO_3_. In the calcium and potassium interaction experiment, Ca0, Ca0.5 Ca1, Ca2, Ca4, and Ca8 represent concentrations of CaCl_2_ at 0, 1.5, 3, 6, 12 and 24 mM, respectively. For other ions (P, Mg, Fe, Zn, Mn, B, Mo, Co, and Cu), only the concentration of the corresponding compound was changed in the medium. Three replicates were performed for each treatment. At least six ovules of one replicate were collected for fiber length measurement after 5 or 10 d culture.

### Fiber length and total fiber unit (TFU) measurement

Fiber-bearing ovules were collected from cultured ovules treated with different ions and used for fiber length measurement according to a previous method (Guo *et al.*, 2016). Fiber-bearing ovules were boiled in double-distilled water until the fibers straightened. Fiber length was measured manually with a ruler. Fiber length from eight ovules was measured per culture bottle, and three biological replicates were carried out for each treatment. For fiber length measurement from field-grown plants, three to five bolls from different YZ1 plants were collected at 3, 5, 8, 10, and 12 DPA, and 15 ovules of each boll were selected for fiber length measurement. The total fiber unit (TFU) was used to determine the fiber yield of wild-type and Ri16 cotton cultured under K0 and K1 conditions. The TFU measurement was performed as described previously (Tan *et al.*, 2013).

### Ionome quantification with ICP-MS

To quantify the content of each ion in ovules and fibers at different stages, samples were collected at 0 to 25 DPA from four cotton varieties grown in the field, *G. hirsutum* (*Gh*) TM-1, Xuzhou142, *xu142-fl*, and *G. barbadense* (*Gb*) 3–79. At 0 DPA, ovules from 200 flowers were collected for each sample. For samples at 3 and 5 DPA, fiber-bearing ovules from 100 bolls were collected. At 10, 15, 20, and 25 DPA, at least 20 bolls were collected from the same position on the plant, with each boll being collected from a different plant. For samples from 5 to 25 DPA, fibers were gently knocked off the ovules with a pestle in liquid nitrogen, and fibers and ovules were separately ground into powder. To analyse changes in ion contents in fiber-bearing ovules treated with different levels of K^+^ or Ca^2+^, fiber-bearing ovules cultured for 10 d with K0 (2 mM K^+^), K1 (27 mM K^+^), and K2 (52 mM K^+^), and fiber-bearing ovules cultured for 5 d with Ca0-K0.5 (0 mM Ca^2+^ and 27 mM K^+^), Ca1-K0.5 (3 mM Ca^2+^ and 27 mM K^+^), and Ca8-K0.5 (24 mM Ca^2+^ and 27 mM K^+^) were collected and washed with double-distilled water. After removing excess water with filter paper, samples were ground into powder with liquid nitrogen. Powder samples were dried in a vacuum freeze-drier (LABCONCO FreeZone®2.5, USA) until constant weight was achieved and analysed for ion contents according to the method described previously by Yang *et al.* (2014). A sample of 0.2 g dry mass was digested with 65% nitric acid in a MARS6 microwave (CEM MARS 6, USA) at a temperature gradient of 120–180 °C for 45 min. After the sample was completely digested, the nitric acid was evaporated at 160 °C for 40 min, and the residue was diluted with deionized water. The metal content of the sample was determined by inductively coupled plasma–mass spectrometry (ICP-MS; Agilent 7700 series, USA).

### RNA extraction and gene expression analysis

After fiber-bearing ovules had been cultured with different levels of exogenous K^+^ or Ca^2+^ for 5 or 10 d, all samples were collected and the fibers were gently removed with a pestle in liquid nitrogen. The fibers and ovules were then separately ground into powder, and 0.1 g samples were used for total RNA extraction according to the method described previously by Zhu *et al.* (2005). cDNA was synthesized with SuperScript III reverse transcriptase (Invitrogen, Carlsbad, CA, USA). qRT-PCR was performed as previously described using an Applied Biosystems 7500 Real-Time PCR System (Guo *et al.*, 2016), and all primers are listed in Supplementary Table S1. *GhUB7* (DQ116411) was used as the internal control to normalize gene expression levels.

### RNA-seq for screening of differentially expressed genes

Fibers cultured for 10 d with three different levels of K^+^ (K0, K1, and K2) and fibers cultured for 5 d with three different levels of Ca^2+^ (K0.5-Ca0, K0.5-Ca1, and K0.5-Ca8) were used for total RNA extraction. Each treatment included two biological replicates, and each biological replicate included six repeats. Twelve samples of total RNA were sent to the BGI Company (Wuhan, China) for RNA-Seq quantification analysis using an Illumina HiSeq^TM^2000. The coding sequence (CDS) and genome sequences (BioProject ID: PRJNA248163) of *G. hirsutum* TM-1 were used as the reference. For each sample there were 12 million total reads. Clean RNA-Seq reads were mapped to the *G. hirsutum* genome and the uniquely mapped reads were extracted for estimation of the expression levels of genes using Cufflinks (Trapnell *et al.*, 2012). Correlation values based on fragments per kilobase of transcript per million mapped reads (FPKM) between two biological replicates of each treatment were higher than 0.92. To identify differentially expressed genes (DEGs) between treatments, pairwise comparisons between two treatments were carried out using the NOIseq method (RPKM value ≥1, fold-change ≥2, and diverge probability ≥0.8) (Tarazona *et al.*, 2011). Gene ontology (GO) terms enrichment analysis of DEGs was carried out using the Blast2GO software with Fisher’s Exact Test. Cluster analysis was performed with Cluster3.0 software.

### Quantification of endogenous hormone with liquid chromatography–electrospray ionization–tandem mass spectrometry

Fiber-bearing ovules cultured for 5 d under Ca0-K0.5, Ca1-K0.5, Ca8-K0.5, Ca0-K1, Ca1-K1, and Ca8-K1 treatments were used for the measurement of abscisic acid (ABA) and jasmonic acid (JA) contents. Fibers or ovules were ground into powder in liquid nitrogen. Samples of 0.1 g were extracted with 750 µl cold extraction buffer (methanol:water:acetic acid, 80:19:1, v/v/v) supplemented with 10 ng ml^−1 2^H_6_-ABA and 10 ng ml^−1^ dihydrojasmonic acid (dh-JA) as internal standards, and shaken on a shaker overnight at 4 °C in the dark. After centrifugation at 12 000 *g* for 15 min at 4 °C, the supernatant was transferred to a new 2-ml centrifuge tube. The precipitate was suspended in 450 µl 80% (v/v) methanol, shaken at 4 °C for 4 h, and tubes were centrifuged to ensure the extracted sample mixed with the remaining supernatant. After the mixed supernatant had been air-dried, the residues were dissolved in 300 µl 10% (v/v) methanol. The solution was filtered through a 0.22-µm nylon membrane (Nylon 66; Jinteng Experiment Equipment Co., Ltd, Tianjing, China) and the hormone was detected using LC-GC/MS according to the method reported previously by Liu *et al.* (2012).

### ROS detection with 2’,7’-dichlorodihydrofluorescein diacetate (2’,7’-DCFDA)

Ovules cultured in medium with different levels of K^+^ or Ca^2+^ for 1, 2, 3, or 4 d were incubated in 0.01 M PBS buffer (135 mM NaCl, 2.7 mM KCl, 1.5 mM KH_2_PO_4_, and 8 mM K_2_HPO_4_, pH 5.2) containing 10 µM 2’,7’-DCFDA (Sigma, D6883, USA). The 2’,7’-DCFDA was dissolved in dimethyl sulfoxide (DMSO) for 30 min in the dark at 30 °C in advance (Guo *et al.*, 2016). After incubation, ovules were washed with double-distilled water for 5 min before imaging. Fluorescence images were obtained using stereo fluorescence microscopy (LEICA MZFLIII, Germany). Dye excitation was at 488 nm; emitted light was detected at 522 nm. More than 10 ovules for each treatment were analysed.

## Results

### Macro-elements preferentially accumulate in fast-elongating fibers

**Fig. 1.**
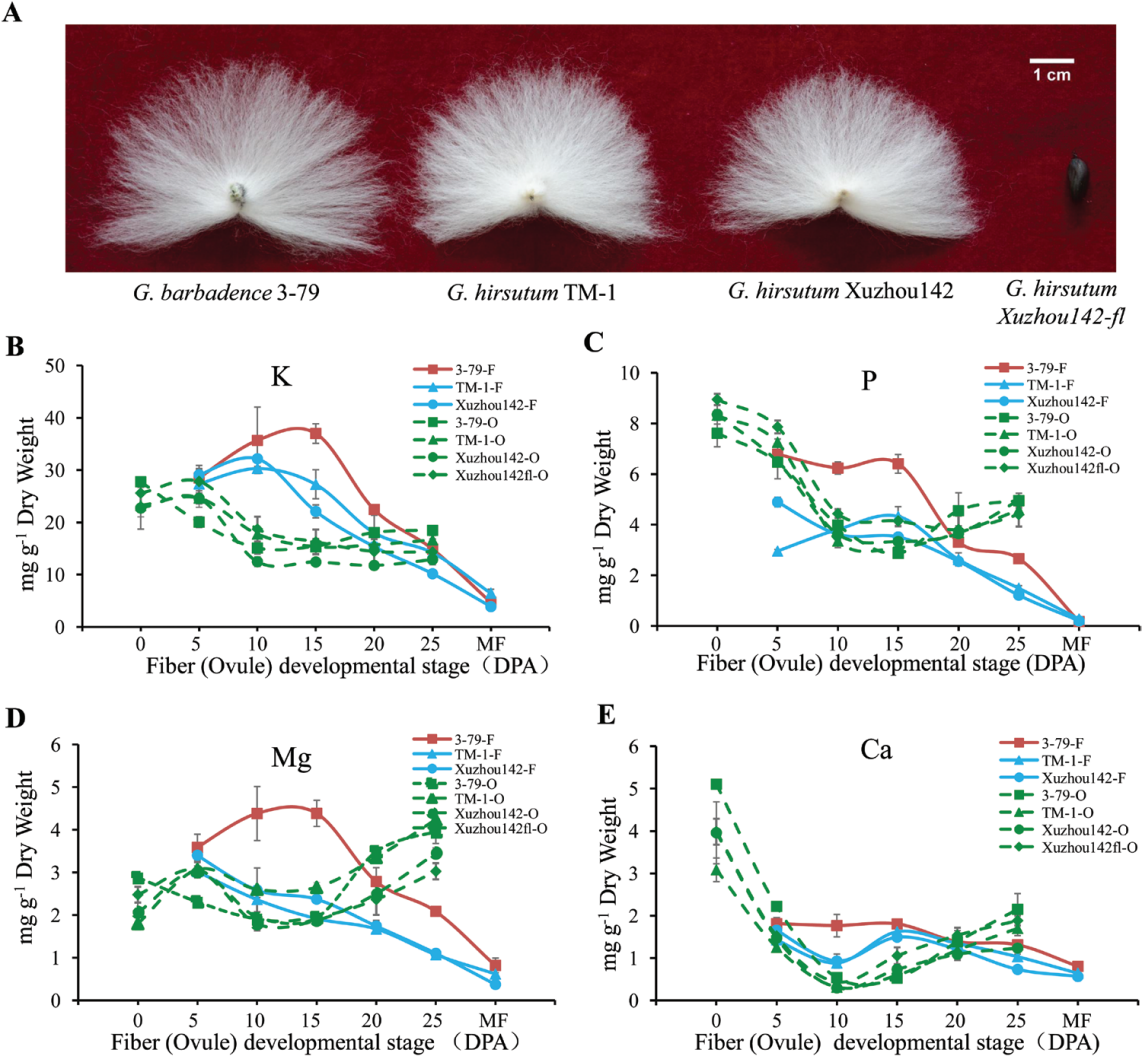
Ionome quantification by inductively coupled plasma mass spectrometry (ICP-MS) in ovules and fibers at different development stages. (A) Four varieties of cotton used for ICP-MS. (B–E) The contents of (B) K, (C) P, (D) Ca, and (E) Mg in ovules and fibers from 0 d post-anthesis (DPA) to the mature stage (MF, mature fiber). Data are means ±SD, *n*=3, three biological repeats. O indicates ovules, F indicates fibers.

To capture the dynamic changes in the ionome of cotton ovules and fibers during different development stages, four cotton varieties [*G. barbadense* (*Gb*) 3–79, and *G. hirsutum* (*Gh*) TM-1, Xuzhou 142, and *Xuzhou 142-fl*; Fig. 1A] were used to quantify the total content of mineral elements using ICP-MS technology. The results showed that the contents of essential elements in the fibers were higher than in ovules during fiber elongation, especially for macro-elements (Fig. 1B–E and Supplementary Fig. S2). During the development of fibers, the contents of K, P, and Mg increased and reached their highest levels at the fast-elongating stage (5–15 DPA). Thereafter, the contents decreased at the secondary cell wall thickening stage, and declined to their lowest levels in the mature fiber (Fig. 1B–D).

### High accumulation of macro-elements, but not Ca^2+^, is associated with longer fibers

Fibers from *Gb* are more desirable than those from *Gh* as they are much longer, finer, and stronger (Supplementary Table S2). At the same fiber development stage, the contents of macro-elements (K, P, and Mg) in *Gb* fibers were higher than those in *Gh* (Fig. 1B–D). In *Gh* at 10 DPA, the macro-element content, especially that of K^+^, reached a maximum in the fibers (K^+^: 30.33 ± 0.38 mg g^−1^ DW to 32.18 ± 0.52 mg g^−1^ DW) and a minimum in the ovules (K^+^: 17.76 ± 0.67 mg g^−1^ DW to 12.47 ± 0.90 mg g^−1^ DW). After 10 DPA, contents of K, P, and Mg began to decrease in fiber cells and increase in ovules (Fig. 1B–D). This suggests that 10 DPA is a key transition point at which ions move from ovule cells to fiber cells during *Gh* fiber development. However, this transition point was delayed to 15 DPA in *Gb*, and the contents of K, P, and Mg in *Gb* fibers increased to 15 DPA, suggesting that there is a longer period of macro-element (K, P, and Mg) accumulation in *Gb* than in *Gh* (Fig. 1B–D).

The pattern of Ca^2+^ accumulation in fibers and ovules was different to that of K, P, and Mg. In *Gb* and *Gh* ovules, the Ca^2+^ content was at a maximum at 0 DPA and decreased to the lowest level at 10 DPA, and then increased subsequently (Fig. 1E). In leaves, Ca^2+^ was the most abundant element (Supplementary Table S3), but in fibers its content was the lowest among the macro-elements, and it did not show a significant change during fiber elongation (Fig. 1E). In *Gh* fibers, the Ca^2+^ content declined from 5 to 10 DPA, then increased at 15 DPA, and finally decreased to the mature fiber stage (Fig. 1E).

### Macro- and micro-elements are necessary for fiber elongation *in vitro*

**Fig. 2.**
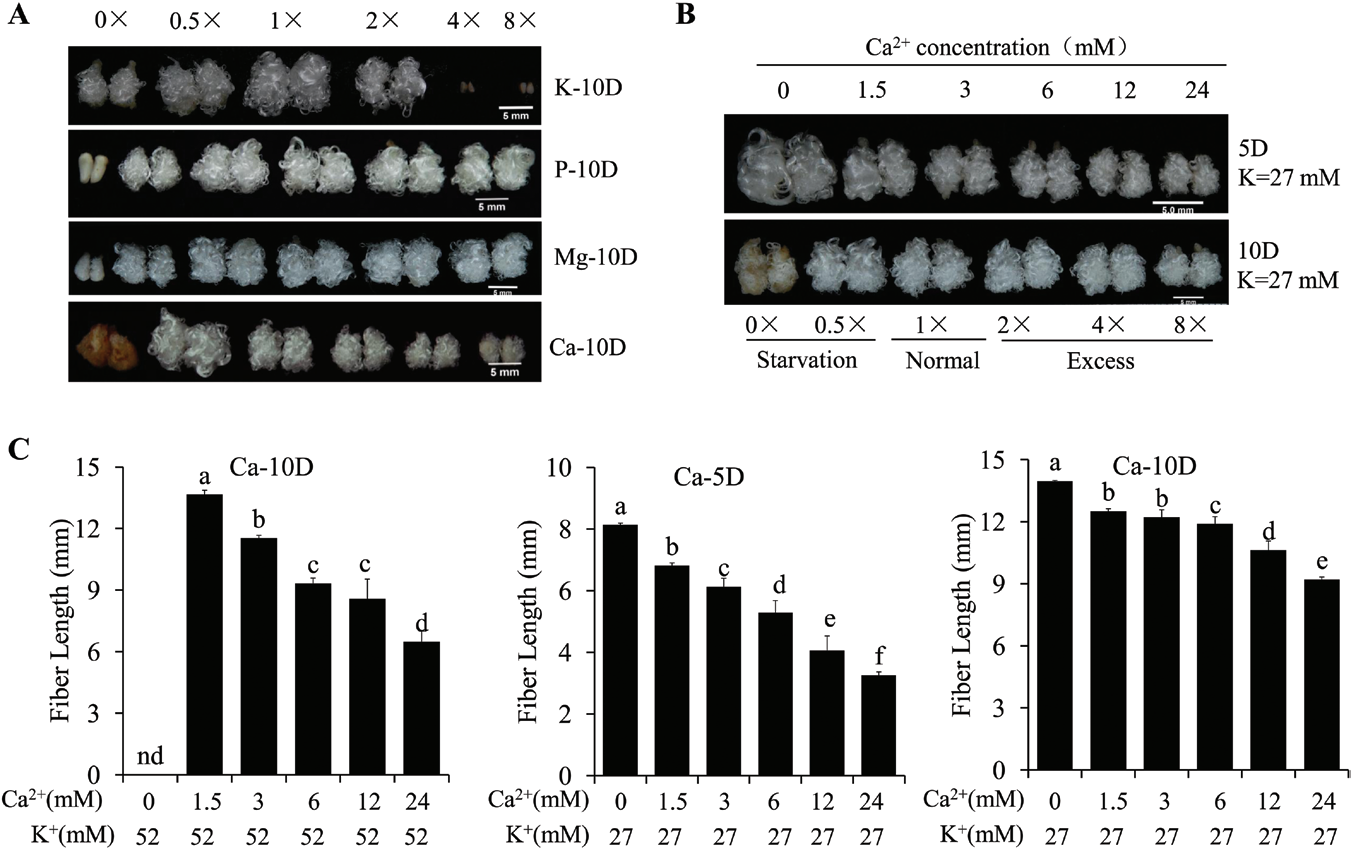
Effects of K, P, Ca, and Mg at different concentrations (0, 0.5-, 1-, 2-, 4-, and 8-fold standard concentration) on development of cultured ovules and fibers. (A) Phenotypes of fiber-bearing ovules cultured with six concentrations of K, P, Ca, or Mg after 10 d culture. Scale bars =5 mm. (B) Phenotypes of fiber-bearing ovules cultured for either 5 d (5D) or 10 d (10D) at six different concentrations of Ca^2+^ under either half-K^+^ or sufficient K^+^ condition. Scale bars =5 mm. (C) Fiber lengths of ovules cultured with different concentrations of Ca^2+^ or K^+^ at either 5 d (5D) or 10 d (10D). Data are means ± SD, *n*=3, three biological replicates. 1× indicates the standard concentration of Ca^2+^ in the culture medium, 0× indicates Ca^2+^ was absent from the medium, and 0.5×, 2×, 4×, and 8× indicate that Ca^2+^ was present at 0.5-, 2-, 4-, and 8-fold that of the standard concentration.

Ovule culture is an excellent system to investigate the molecular mechanisms that control fiber initiation and elongation (Beasley and Ting, 1973). At the early elongation stage, the lengths of field-grown and ovule-cultured fibers showed little difference until 10 DPA (Supplementary Fig. S3). To determine the effects of specific ions on fiber elongation, we used an *in vitro* ovule culture system. A series of concentrations (0, 0.5-, 1-, 2-, 4-, and 8-fold compared to the basic concentration) for each of 11 essential elements (K, P, Ca, Mg, Fe, Zn, Mn, B, Mo, Co, and Cu) was set up. After 5 or 10 d culture, fiber phenotypes and lengths were determined (Fig. 2 and Supplementary Fig. S4).

Lack of K, P, Mg, Zn, or Mn inhibited fiber elongation (Fig. 2A and Supplementary Fig. S4A–C). P deficiency not only completely inhibited fiber elongation, but also suppressed ovule development, which resulted in a smaller and naked ovule (Fig. 2A and Supplementary Fig. S4B). Ca^2+^ or Fe deficiency suppressed ovule and fiber development and induced tissue browning (Fig. 2A and Supplementary Fig. S4B, C).

Application of high concentrations of P, Mg, or Mn in the BT growth medium did not affect fiber elongation significantly, but high application of K, Ca, Zn, or Fe significantly altered the pattern of fiber elongation (Fig. 2A and Supplementary Fig. S4A–C). A 2-fold concentration of K^+^ in the medium suppressed fiber elongation, while a 4- or 8-fold concentration led to ovule death (Fig. 2A and Supplementary Fig. S4B). When an 8-fold Zn concentration was applied, development was inhibited, resulting in very small ovules lacking fibers. For the essential element Fe, a 2-fold concentration (60 µM) in the medium was the optimum level to maintain fiber elongation (Supplementary Fig. S4A–C). Alterations in exogenous concentrations of B, Mo, Co, and Cu had no obvious effect on fiber development (Supplementary Fig. S4).

### Integration of Ca^2+^ deficiency and reduced K^+^ limits tissue damage and produces longer fibers

Ca^2+^ was the macro-element with the lowest content in fibers (Fig. 1E). The macro-element K^+^ had the highest content compared to other essential elements, and the content was also higher in elongating fibers than in ovules at the same developmental stage (Fig. 1B). Given that Ca^2+^ and K^+^ are important for normal fiber development (Han *et al.*, 2014; Tang *et al.*, 2014), we aimed to understand the nature of the interaction between Ca^2+^ and K^+^ to produce longer fibers.

Under normal K^+^ supply (52 mM, K1) in the ovule culture system, Ca^2+^ deficiency induced ovule and fiber browning (a sign of tissue damage) (Fig. 2A), while a reduced level of Ca^2+^ in the medium (1.5 mM, Ca0.5) produced longer fibers (13.65 ± 0.22 mm) than the normal Ca^2+^ concentration (3 mM, Ca1) (11.53 ± 0.14 mm) (Fig. 2C). After 5 or 10 d culture under half-K^+^ (27 mM, K0.5) conditions, the length of fibers cultured under Ca^2+^ deficiency (0 mM Ca^2+^ and 27 mM K^+^, Ca0-K0.5) was longer than when treated with standard Ca^2+^ and half-K^+^ (3 mM Ca^2+^ and 27 mM K^+^, Ca1-K0.5), i.e. 8.13 ± 0.05 mm compared to 6.13 ± 0.27 mm (5 d culture) and 13.96 ± 0.03 mm compared to 12.22 ± 0.36 mm (10 d culture) (Fig. 2B, C). Fibers treated with Ca^2+^ deficiency and half-K^+^ (0 mM Ca^2+^ and 27 mM K^+^, Ca0-K0.5) started to show browning by the 10th day (Fig. 2B). The different phenotypes of fibers cultured with different concentrations of Ca^2+^ and K^+^ indicated that a combination of simultaneously reduced Ca^2+^ and K^+^ produces longer fibers at the early elongating stage.

### Reduced K^+^ induces up-regulation of ROS scavenging-related genes

**Fig. 3.**
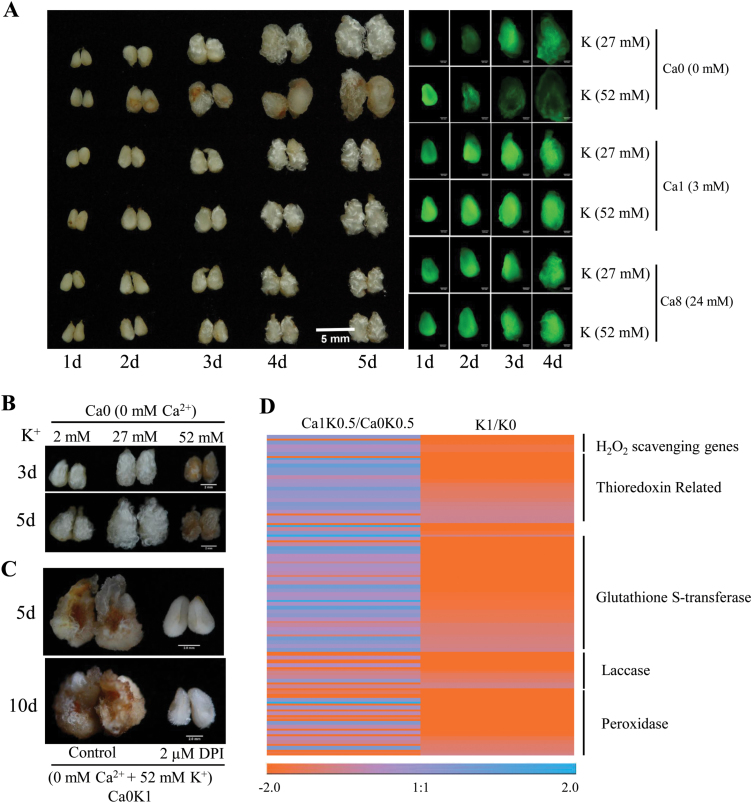
Reduced K^+^ in the growth medium increases ROS-scavenging ability and inhibits the development of tissue browning. (A) Phenotypes (left) and ROS levels (right) of fiber-bearing ovules cultured under different concentrations of K^+^ or Ca^2+^ for up to 5 d. Scale bar =5 mm. ROS levels were detected by staining with the fluorescent dye 2’,7’-DCFDA (10 µM). (B) Phenotypes of ovules and fibers cultured with different concentrations of K^+^ under Ca^2+^-deficient condition for 3 or 5 d. Scale bars =5 mm. (C) Application of 2 µM DPI (an inhibitor of the ROS generation enzyme RBOH oxidase) inhibited Ca^2+^ deficiency-induced browning after 5 or 10 d culture in medium containing 0 mM Ca^2+^ and 52 mM K^+^. Scale bars =2 mm. (D) Cluster analysis of ROS-scavenging genes in response to reduced K^+^ or Ca^2+^ deficiency based on the RPKM values from RNA-seq analysis. Ca1K0.5 = 3 mM Ca^2+^ and 27 mM K^+^; Ca0K0.5 = 0 mM Ca^2+^ and 27 mM K^+^; K1 = 52 mM K^+^; K0 = 2 mM K^+^.

Ca^2+^ deficiency-promoted fiber elongation was found to depend on the concentration of K^+^ in the medium. Under Ca^2+^ deficiency, a sufficient concentration of K^+^ (52 mM, K1) did not enhance fiber elongation, but instead resulted in a brown ovule with suppressed fiber development; however, reduced K^+^ (27 mM) alleviated Ca^2+^ deficiency-induced stress (Fig. 2A–C). To further investigate this, we carried out a continuous culture series of six treatments (Ca0-K0.5, Ca1-K0.5, Ca8-K0.5, Ca0-K1, Ca1-K1, and Ca8-K1) over 5 d. At the second day, browning appeared in ovules in Ca0-K1 (0 mM Ca^2+^ and 52 mM K^+^), which became more obvious after 5 d culture (Fig. 3A). Ovules and fibers treated with Ca0-K0.5 (0 mM Ca^2+^ and 27 mM K^+^) did not brown, and the fibers were longer than in Ca1-K0.5 (3 mM Ca^2+^ and 52 mM K^+^) (Fig. 3A). If the concentration of K^+^ was decreased to 2 mM under Ca^2+^-deficient conditions (Ca0-K0, 0 mM Ca^2+^ and 2 mM K^+^), the brown phenotype in the ovules was delayed, but fiber elongation was inhibited (Fig. 3B). These results indicate that reduced K^+^ in the growth medium compensates for Ca^2+^ deficiency-induced stress that results in browning.

We hypothesized that the browning induced by Ca^2+^ deficiency under sufficient K^+^ conditions was caused by ROS-induced cell death (Maathuis, 2009). ROS levels were measured in ovules, and were higher in Ca0-K1 (0 mM Ca^2+^ and 52 mM K^+^) than in Ca0-K0.5 (0 mM Ca^2+^ and 27 mM K^+^) or Ca1-K1 (3 mM Ca^2+^ and 52 mM K^+^) at the first day (Fig. 3A). As the culture time progressed, ROS levels decreased gradually in ovules in Ca0-K1 (0 mM Ca^2+^ and 52 mM K^+^) and were lower in this treatment than in Ca0-K0.5 (0 mM Ca^2+^ and 27 mM K^+^) at the fourth day (Fig. 3A). When 2 µM DPI (the inhibitor of the ROS generation enzyme RBOH oxidase) was added to the Ca0-K1 medium, the browning was significantly inhibited (Fig. 3C). This suggests that the browning phenotype under sufficient K^+^ in Ca^2+^-deficient medium (0 mM Ca^2+^ and 52 mM K^+^) may derive from the uncontrolled accumulation of ROS.

Reduced concentrations of K^+^ (2 or 27 mM K^+^) in the growth medium delayed Ca^2+^ deficiency-induced browning. To test whether this might be due to increased ROS-scavenging ability, we performed RNA-seq analysis to screen genes that respond to Ca^2+^ deficiency or to reduced K^+^ (Supplementary Fig. S5). Blast2GO analysis of up-regulated genes in K0 (2 mM K^+^) fibers revealed that oxidoreductase-related processes are significantly enriched (Supplementary Table S4). ROS-scavenging genes involved in the response to reduced K^+^ or Ca^2+^ deficiency were clustered (Fig. 3D). Among the 206 identified genes, 187 were up-regulated in K0 fibers compared to K1 (52 mM K^+^), including the anti-oxidative genes *LAC*, *POD*, *GST*, *APX*, *GPX*, and *MT* (Supplementary Table S5). Marker genes induced by low K^+^ that are involved in ROS signaling, such as *RCI3*, *RBOHD*, and *GPX6*, were also identified in K0 fibers. Among 61 up-regulated glutathione S-transferase (GST) genes in K0 fibers, 37 were the *Tau*-type *GST* (Supplementary Table S5). However, the number of ROS-scavenging genes in fibers treated with Ca^2+^ deficiency (63) was less than the number in fibers treated with reduced K^+^ (187) (Fig. 3D). These results suggest that reduced K^+^ induced ROS-scavenging ability to alleviate Ca^2+^ deficiency-induced browning.

### Reduced K^+^ availability maintains ABA and JA levels to suppress Ca^2+^ deficiency-induced ROS activity

**Fig. 4.**
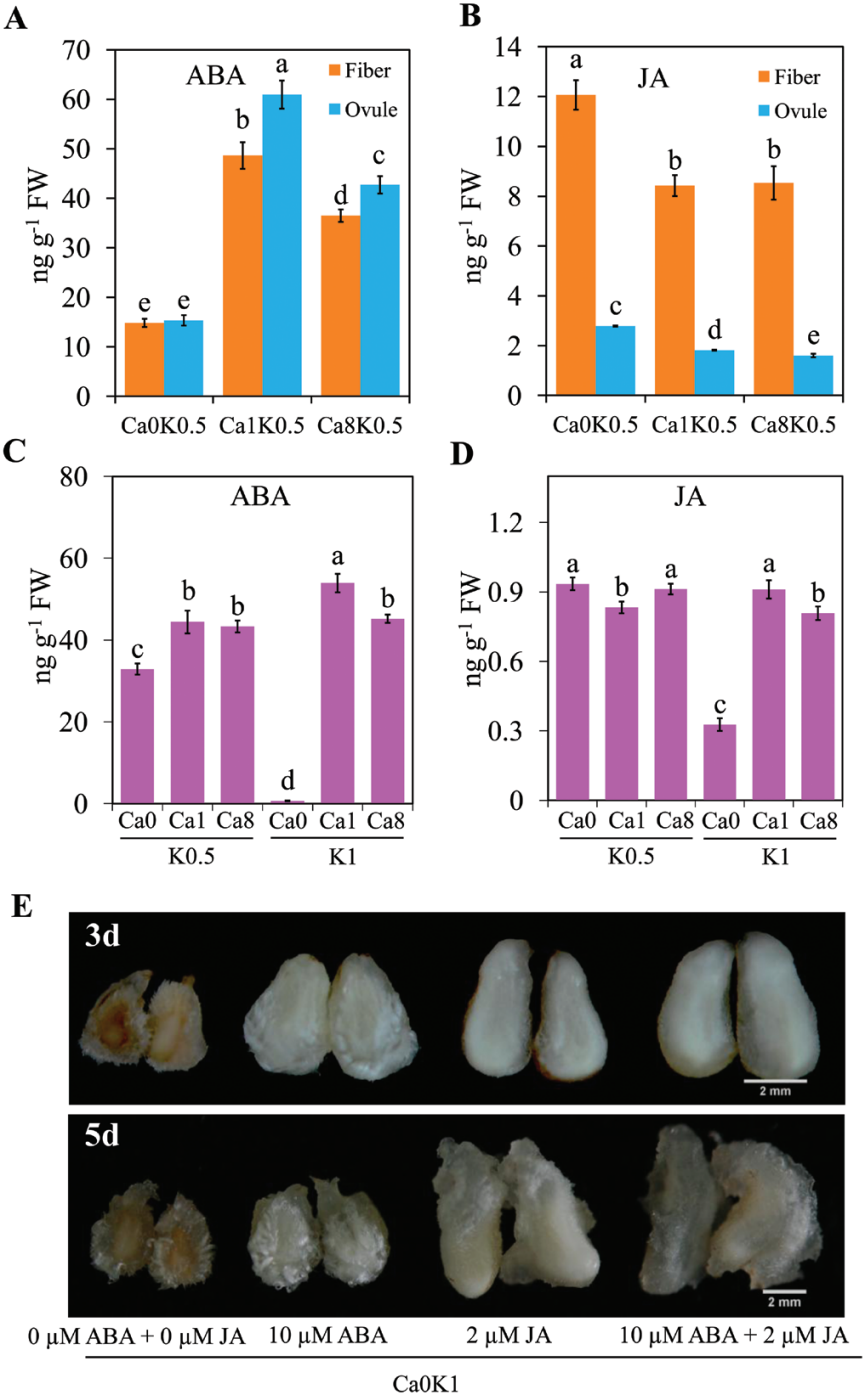
Reduced K^+^ in the growth medium alleviates Ca^2+^ deficiency-induced browning, associated with ABA or JA signaling. (A, B) Levels of ABA (A) and JA (B) in ovules and fibers cultured for 5 d under three concentrations of Ca^2+^ with reduced K^+^. (C, D) Levels of ABA (C) and JA (D) were quantified in fiber-bearing ovules cultured for 5 d with different concentrations of Ca^2+^ and K^+^. Different letters above the bars indicate differences are significant at *P*<0.05 (ANOVA and Duncan’s multiple comparisons). Data are means ±SD of four biological replicates. (E) Application of exogenous ABA and/or JA alleviated Ca^2+^ deficiency-induced browning in ovules. Scale bars =2 mm. Treatments are as follows: Ca0, 0 mM Ca^2+^; Ca1, 3 mM Ca^2+^; Ca8, 24 mM Ca^2+^; K0.5, 27 mM K^+^; K1, 52 mM K^+^.

In addition to enhancing ROS-scavenging ability, reduced K^+^ supply may activate other signaling systems to protect against damage by Ca^2+^ deficiency. Therefore, endogenous hormones were quantified in ovules and fibers cultured with different levels of K^+^ or Ca^2+^. After 5 d culture, the level of ABA was decreased and JA was increased in ovules and fibers cultured in Ca0-K0.5 (0 mM Ca^2+^ and 27 mM K^+^), compared to Ca1-K0.5 (3 mM Ca^2+^ and 27 mM K^+^) (Fig. 4A, B). ABA and JA levels were also determined in fiber-bearing ovules after 5 d of culture with three levels of Ca^2+^ under half-K^+^ or K^+^-sufficient conditions. ABA was undetectable in Ca0-K1 (0 mM Ca^2+^ and 52 mM K^+^) ovules (Fig. 4C), and this was associated with tissue browning. However, the levels of JA also significantly decreased in Ca0-K1 ovules compared with Ca1-K1 (3 mM Ca^2+^ and 52 mM K^+^) or Ca0-K0.5 ovules (Fig. 4D). These results suggest that reduced K^+^ availability maintains ABA levels and increases JA, and this is associated with the alleviation of ROS damage induced by Ca^2+^ deficiency.

To test this possibility, we carried out ovule culture in the presence of either 10 µM ABA, 2 µM JA, or 10 µM ABA plus 2 µM JA in Ca0-K1 medium, and monitored the browning phenotype. After 3–5 d culture, application of exogenous JA or ABA, or both together, could significantly alleviate Ca^2+^ deficiency-induced browning in ovules and fibers (Fig. 4E). Application of exogenous ABA not only suppressed browning, but also partially rescued fiber development. Furthermore, the application of JA suppressed browning and completely inhibited fiber development (Fig. 4E). After 5 d culture in Ca0-K1 medium with 2 µM JA or 10 µM ABA plus 2 µM JA, the ovules formed calluses (Fig. 4E).

### Ca deficiency up-regulates the fiber cell loosening-related genes *PSK*, *EXP*, and *XTH*

**Fig. 5.**
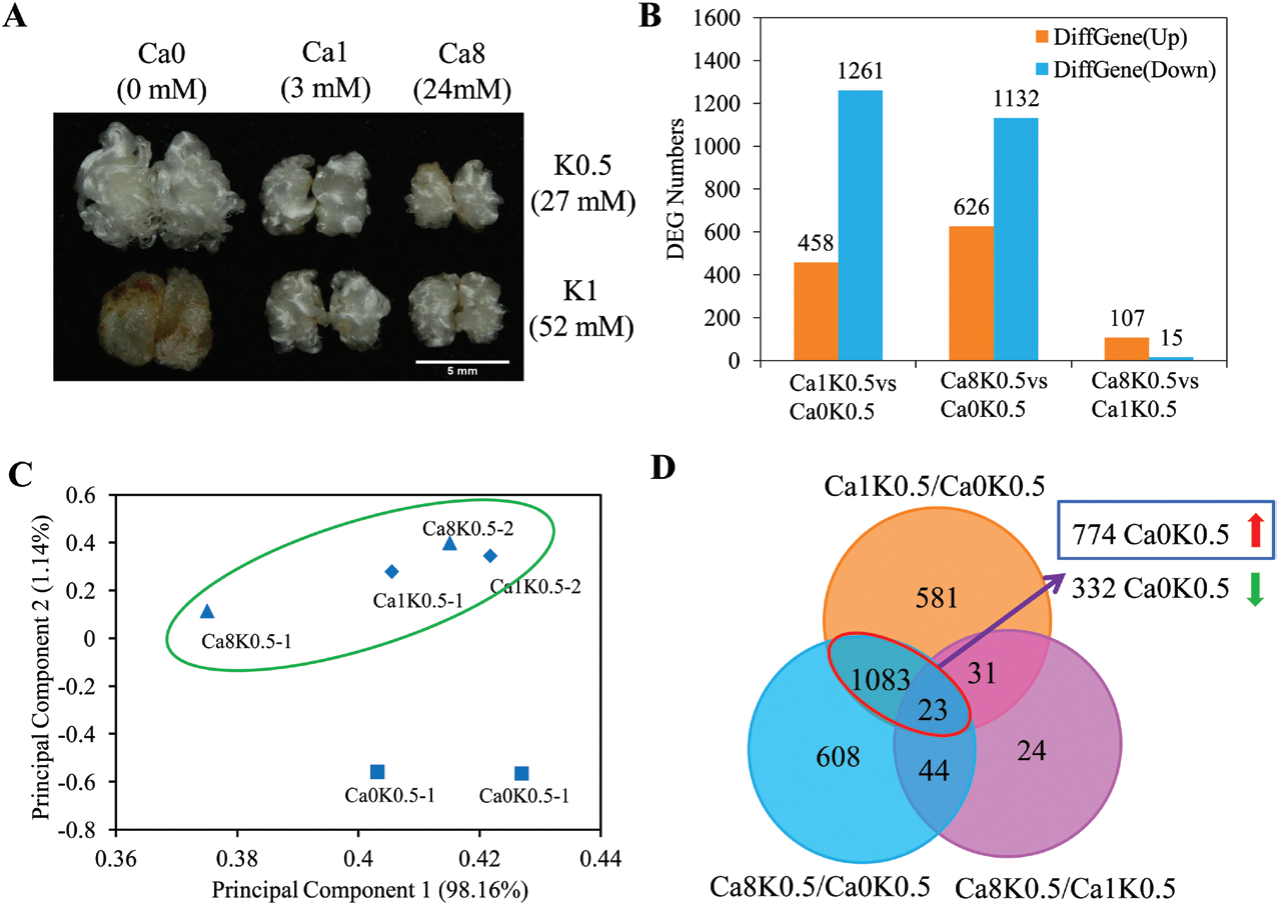
Analysis of differentially expressed genes (DEGs) in fibers cultured with three Ca^2+^ concentrations for 5 d under half-K^+^ (K0.5) or K-sufficient (K1) conditions. (A) Phenotypes of fiber-bearing ovules. Scale bar =5 mm. (B) DEGs in fibers cultured half-K^+^ conditions. (C) Principal component analysis of six samples treated with different levels of Ca^2+^ under K0.5 condition based on gene expression levels. (D) Venn diagram showing overlapping DEGs in the three comparisons indicated. Treatments: K0.5, 27 mM K^+^; K1, 52 mM K^+^; Ca0, 0 mM Ca^2+^; Ca1, 3 mM Ca^2+^; Ca8, 24 mM Ca^2+^.

Ca^2+^ deficiency promoted fiber elongation under reduced K^+^ conditions (Fig. 5A). To elucidate the mechanism, we collected fibers treated with three levels of Ca^2+^ under half-K^+^ conditions (Ca0-K0.5, Ca1-K0.5, and Ca8-K0.5) for RNA-seq analysis and identified genes involved in the Ca^2+^-deficiency response. A total of 3599 differentially expressed genes (DEGs) were identified (fold change ≥2, RPKM≥1) among the different treatments (Fig. 5B). Principal component analysis (PCA) showed that Ca^2+^ deficiency-treated samples were clustered away from the samples receiving normal or excess Ca^2+^ (Fig. 5C), showing that Ca^2+^ deficiency induces several specific genes/pathways.

**Fig. 6.**
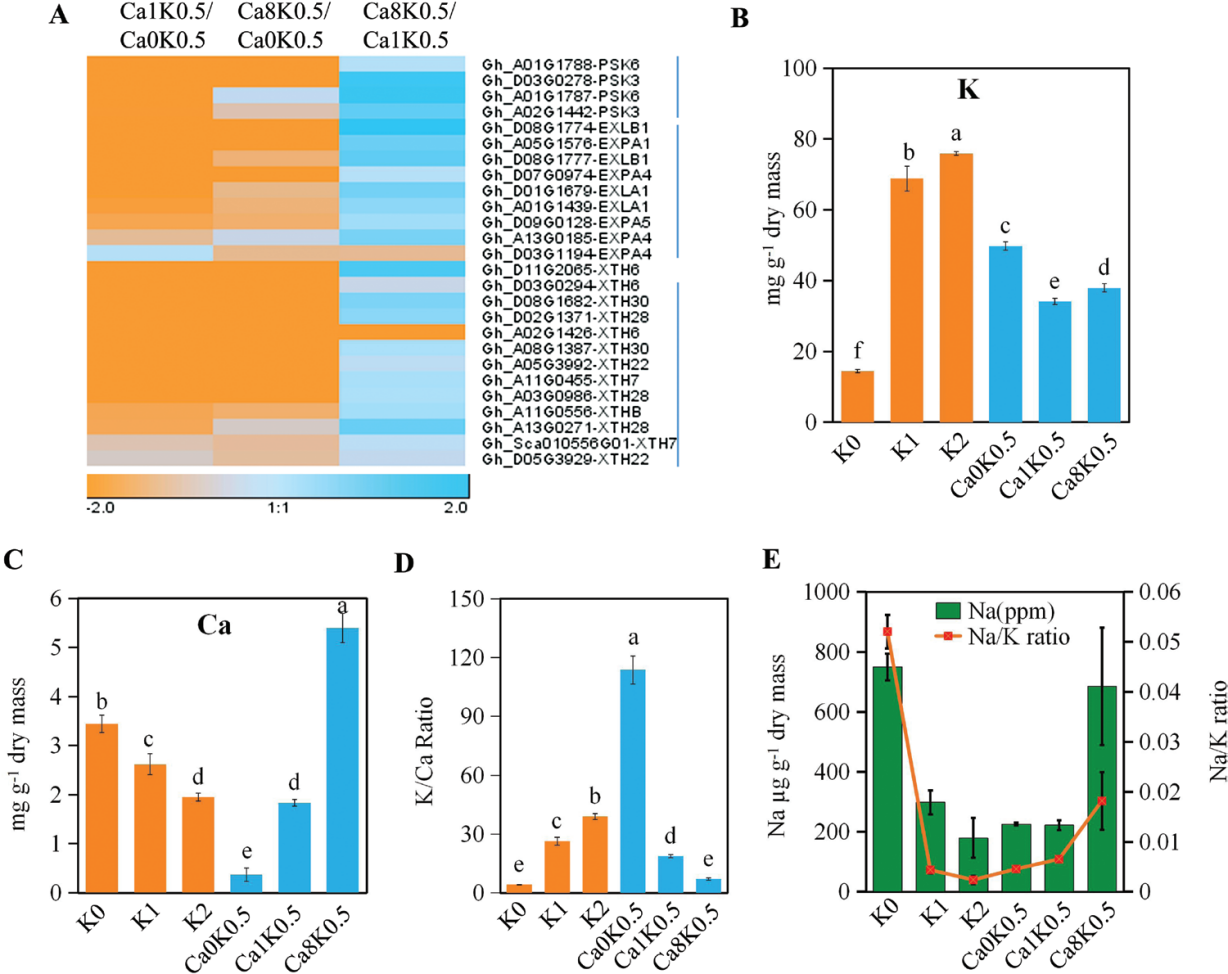
Ca^2+^ deficiency up-regulates the expression of *PSK*, *EXP*, and *XTH* and increases K^+^ uptake. (A) Cluster analysis of expression levels of *PSK*, *EXP*, and *XTH* genes in fiber-bearing ovules treated with different levels of Ca^2+^ under K0.5 conditions. (B) Quantification of K^+^ in fiber-bearing ovules cultured with different levels of K^+^ and Ca^2+^ in the growth medium. (C) Quantification of Ca^2+^ in fiber-bearing ovules cultured with different levels of K^+^ and Ca^2+^ in the medium. (D) K^+^/Ca^2+^ ratios in fiber-bearing ovules cultured with different concentrations of K^+^ and Ca^2+^ in the medium. (E) Na^+^ contents and Na^+^/K^+^ ratios in fiber-bearing ovules cultured with different levels of K^+^ or Ca^2+^ in the medium. Data are means ±SD of three biological repeats. Different letters above bars indicate differences are significant at *P*<0.05 (one-way ANOVA and Duncan’s multiple comparisons).Treatments: K0, 2 mM K^+^; K0.5, 27 mM K^+^; K1, 52 mM K^+^; K2, 102 mM K^+^; Ca0, 0 mM Ca^2+^; Ca1, 3 mM Ca^2+^; Ca8, 24 mM Ca^2+^.

To identify specific pathways related to the fiber elongation, GO category analysis was performed with the 1262 genes up-regulated in Ca0-K0.5 compared to Ca1-K0.5. The cellular components ‘extracellular region’, ‘apoplast’ and ‘cell wall’, and the biological processes ‘xyloglucan:xyloglucosyl transferase activity’ and ‘hydrolase activity’ were significantly enriched (Supplementary Table S6). The same categories were also enriched in the 1132 up-regulated genes in Ca0-K0.5 compared to Ca8-K0.5 (Supplementary Table S7). Among the overlapping 1106 DEGs between the comparison of Ca1K0.5/Ca0K0.5 and Ca8K0.5/Ca0K0.5, 774 genes were induced by Ca^2+^ deficiency (Fig. 5D). Of these, 774 up-regulated genes were also involved in the functional categories ‘extracellular region’, ‘apoplast’, ‘cell wall’, ‘xyloglucan:xyloglucosyl transferase activity’, and ‘hydrolase activity’ (Supplementary Table S8). Further analysis showed that the genes related to these terms were mainly divided into three types, namely *PSK* (phytosulfokine), *EXP* (expansin), and *XTH* (xyloglucan endotransglycosylases/hydrolases) (Fig. 6A). According to previous reports, these genes play positive roles in fiber elongation (Lee *et al.*, 2010; Han *et al.*, 2014; Li *et al.*, 2016). We confirmed that these genes were significantly induced by Ca^2+^ deficiency under reduced or sufficient K^+^ conditions using qRT-PCR (Supplementary Fig. S6).

### Ca^2+^ deficiency-induced fiber elongation is associated with the K^+^ content

It has been reported that high accumulation of K^+^ in the vacuole provides cells turgor, and PSK can slow down the efflux of K^+^ to promote fiber cell elongation (Maathuis, 2009; Han *et al.*, 2014). To investigate whether faster fiber elongation induced by Ca^2+^ deficiency was associated with K^+^ content, we quantified the ion contents in fiber-bearing ovules cultured with different concentrations of K^+^ or Ca^2+^. With a decrease of K^+^ in the growth medium, its content in the ovule also decreased, while the contents of Ca^2+^ and Na^+^, and the Na^+^/K^+^ ratio increased (Fig. 6B–E). Under half-K^+^ conditions, the reduced Ca^2+^ in the medium led to an increase of K^+^ and a decrease of Ca^2+^ in the ovules (Fig. 6B, C). The K^+^/Ca^2+^ ratio in fiber-bearing ovules in Ca0-K0.5 was significantly increased compared to the ratio in Ca1-K0.5 (Fig. 6D), which suggests that Ca^2+^ deficiency may activate the accumulation of K^+^ in ovules and fiber cells.

### *GhCIPK6* mediates the uptake of K^+^ under Ca^2+^-deficiency conditions

**Fig. 7.**
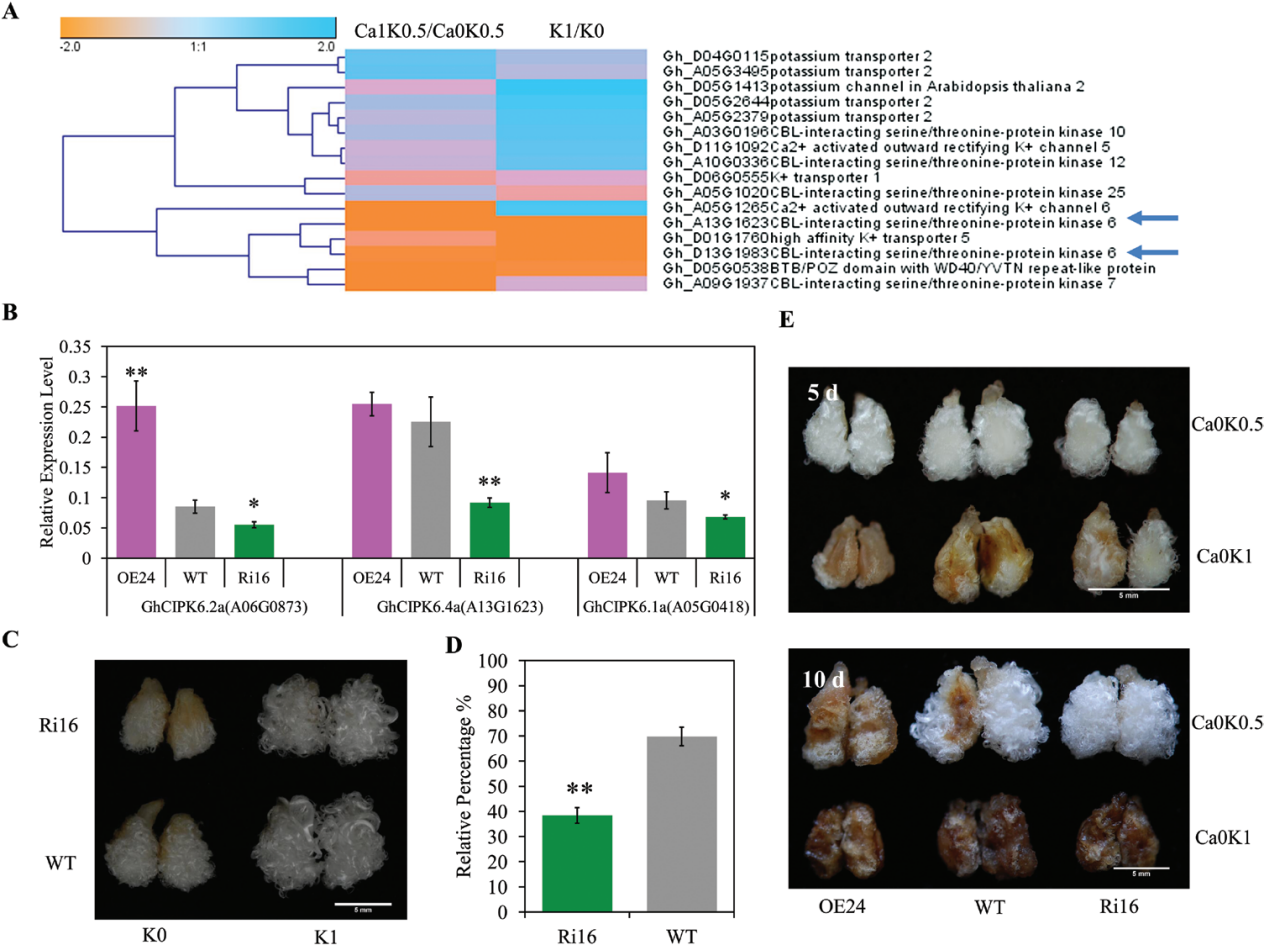
*GhCIPK6* mediates the uptake of K^+^ under Ca^2+^-deficient conditions. (A) Cluster analysis of K^+^ uptake genes in response to reduced K^+^ and Ca^2+^ deficiency based on the RPKM values from RNA-seq analysis. (B) The expression levels of *GhCIPK6* genes in ovules of transgenic cotton at 0 d post-anthesis (DPA). *GhUB7* was used as an internal control to normalize the gene expression level. Data are means ± SD of three biological repeats. OE24, *GhCIPK6.2a(Gh_A06G0873*) 35S overexpressed line; Ri16, *GhCIPK6* RNAi line; WT, wild-type. (C) Phenotypes of the *GhCIPK6* RNAi (Ri16) transgenic line and the wild-type (WT) cotton cultured under reduced (K0) or sufficient (K1) K^+^ conditions. Scale bar =5 mm. (D) Relative percentage of total fiber units (TFU) of Ri16 and WT ovules cultured under reduced (K0) compared to sufficient (K1) K^+^ conditions. Three biological replicates were conducted. (E) Phenotypes of *GhCIPK6* transgenic lines and wild-type (WT) cotton cultured under Ca^2+^-deficient conditions with different K^+^ in the medium for 5 or 10 d. Scale bars =5 mm. Asterisks in (B, C) indicate that differences are significant at *P<0.05, or **P<0.01 (Student’s *t*-test). Treatments: K0, 2 mM K^+^; K0.5, 27 mM K^+^; K1, 52 mM K^+^; Ca0, 0 mM Ca^2+^; Ca1, 3 mM Ca^2+^.

To understand how Ca^2+^ deficiency might induce K^+^ uptake, we analysed changes in the expression levels of marker genes involved in K^+^ uptake, based on the expression profiles in K1/K0 and in Ca1-K0.5/Ca0-K0.5. First, we performed RNA-seq analysis to identify DEGs in fibers cultured for 10 d with three levels of K^+^ in the culture medium (Supplementary Fig. S5). Then, by checking the expression profiles of K^+^ transporters or channels and K^+^ absorption-regulated genes in the reduced K^+^ and Ca^2+^ deficiency treatment, we found four key genes that were induced by both low K^+^ and Ca^2+^ deficiency, namely *Gh_A13G1623* (*CIPK6*), *Gh_D13G1983* (*CIPK6*), *Gh_D01G1760* (*HAK5*), and *Gh_D05G0538* (encoding a BTB/POZ domain with WD40/YVTN repeat-like protein) (Fig. 7A). It has previously been demonstrated that *CBL-INTERACTING PROTEIN KINASE6* (*CIPK6*) interacting with AKT2 (a *Shaker*-type K^+^ channel) controls K^+^ uptake under K^+^-deficient conditions in Arabidopsis (Held *et al.*, 2011).

In *G. hirsutum*, there were eight *CIPK6* genes that showed high similarity to *AtCIPK6* and they were divided into four clades (Supplementary Fig. S7A). *GhCIPK6.3a*(*Gh_A07G0210*) and *GhCIPK6.3d*(*Gh_D07G0265*) expression were undetectable in ovules and fibers, but three other clades of *CIPK6* genes were induced by reduced K^+^ (K0, 2 mM K^+^) or Ca^2+^ deficiency (Supplementary Fig. S7B). Three *GhCIPK6* genes were significantly up-regulated after 6 h treatment in reduced K^+^ (K0) (Supplementary Fig. S7C), consistent with a possible role for *GhCIPK6* in K^+^ uptake.

We constructed transgenic cotton lines either overexpressing *GhCIPK6.2a*(*Gh_A06G0873*) (line OE24) or silencing *GhCIPK6* by RNAi (line Ri16) (Fig. 7B). The transcript levels of all *GhCIPK6* genes were detected in 0 DPA ovules of transgenic and wild-type cotton. Only *GhCIPK6.2a*(*Gh_A06G0873*) was up-regulated in line OE24 and three *GhCIPK6* genes (*GhCIPK6.1*, *GhCIPK6.2*, and *GhCIPK6.4*) were down-regulated in line Ri16 (Fig. 7B). To confirm whether *GhCIPK6* mediated the uptake of K^+^ in ovules and fibers, we checked the response of *GhCIPK6* transgenic cotton to reduced K^+^ treatment (K0) (Fig. 7C). Ionome analysis of fiber-bearing ovules showed that K^+^ contents were significantly lower in the *GhCIPK6*-suppressed line (Ri16) than in the wild-type under Ca^2+^-deficient conditions (Supplementary Fig. S8), confirming a role for *GhCIPK6* in K^+^ uptake. The much lower value of total fiber units (TFU) in the Ri16 line under reduced K^+^ (K0) conditions is consistent with this view (Fig. 7D). In ovules cultured in Ca^2+^-deficient growth medium, the OE24 line showed browning earlier than the wild-type under different K^+^ conditions (K0.5, 27 mM K^+^, and K1, 52 mM K^+^) after 5 or 10 d culture, displaying an increased sensitivity to Ca^2+^ deficiency (Fig. 7E). The *GhCIPK6*-suppressed cotton line (Ri16) on the other hand showed a higher tolerance to Ca^2+^ deficiency than the wild-type (Fig. 7E). These results show the critical role of *GhCIPK6* to alleviate the Ca^2+^ deficiency-induced ROS-mediated browning, through the regulation of K^+^ availability to the developing cotton fibers.

## Discussion

### Ca^2+^ deficiency-induced browning can be partially rescued by reduced K^+^ availability

The cotton fiber cell is a useful model system to study plant cell elongation and secondary cell wall synthesis. To improve our understanding of the role of mineral nutrients in cotton fiber growth and development, we examined the role of the macro-elements K, P, Ca, and Mg and the micro-elements Zn and Fe in fiber elongation. We found that a decrease in Ca^2+^ in fiber cells can promote elongation. Ionome analysis during fiber developmental stages revealed a low content of Ca^2+^ and a high content of K^+^, which suggests that cotton might behave as a calcifuge during fiber elongation (Fig. 1). A recent study has shown that the complete absence of Ca^2+^ in ovule culture medium significantly inhibits fiber elongation, and exogenous the application of relatively low Ca^2+^ (0.5 or 1 mM) has a greater effect in promoting fiber elongation than a more normal higher Ca^2+^ concentration (3 mM) (Zhang *et al.*, 2016). A previous study also reported that application of the Ca^2+^ pool release channel blocker 2-aminoethoxydiphenyl borate (2-APB) inhibits Ca^2+^ influx into fiber cells, which suppresses elongation. Moreover, it also found that Ca^2+^ starvation (0 or 1 mM) promotes early fiber elongation, and excess Ca^2+^ (50 mM) inhibits elongation (Tang *et al.*, 2014). Both results imply the essential role of Ca^2+^ in fiber elongation.

In agreement with this, we found that a decrease in Ca^2+^ in the culture medium promoted fiber elongation (Fig. 2). However, if Ca^2+^ was completely absent from the medium, the effect was different and depended on K^+^ availability. Under Ca^2+^-deficient conditions, sufficient K^+^ (52 mM) induced ovule and fiber browning due to the effects of ROS; however, half-K^+^ (27 mM) not only suppressed tissue browning but also promoted fiber elongation, whilst very low K^+^ (2 mM) inhibited both tissue browning and fiber elongation (Figs 2 and 6B). To check that Ca^2+^ was completely absent in the Ca^2+^-deficient medium, we measured its concentration, and this confirmed that Ca^2+^ was undetectable both before and after culturing with ovules (Supplementary Table S9). To further confirm the effects of the lack of Ca^2+^, we applied the Ca^2+^ chelator ethylene glycol-bis(2-aminoethylether) tetraacetic acid (EGTA) to the ovules treated with different levels of Ca^2+^ or K^+^. After 3 d culture, ovules and fibers treated with 1 mM EGTA showed the same phenotypes as those that were not treated (Supplementary Fig. S9). These results suggest that Ca^2+^ deficiency is a double-edged sword for fiber elongation, on the one hand inducing cell wall loosening to promote fiber elongation, and on the other damaging cells and inducing death. It means that the effect of a decrease in Ca^2+^ promoting faster fiber elongation of early cotton fibers depends on the K^+^ content.

A reduced K^+^ concentration in the growth medium compensated for the damage induced by Ca^2+^ deficiency, leading to maintained fiber growth. Reduced K^+^ increased ROS-scavenging activity and maintained ABA/JA levels, which rescued the browning of ovules and fibers caused by Ca^2+^ deficiency (Figs 3 and 4). In Arabidopsis, ABA can alleviate Fe deficiency-induced leaf chlorosis by releasing Fe from the root cell walls and delivering it to the leaves (Lei *et al.*, 2014). Low K^+^ also activates the JA signaling pathway and the biosynthesis of oxylipins and glucosinolates, which increases tolerance to damage by thrips (Armengaud *et al.*, 2004, 2010; Troufflard *et al.*, 2010). In our study, reduced K^+^ availability increased the levels of JA and maintained the levels of ABA in ovules under Ca^2+^ deficiency (Fig. 4C, D). Application of exogenous JA or ABA to Ca^2+^-deficient medium suppressed the browning of ovules (Fig. 4E).

Based on these results, we conclude that reduced K^+^ availability alleviates Ca^2+^ deficiency-induced browning in part by regulating the ABA or JA signaling pathway, as well as through the regulation of K^+^ uptake.

### Ca^2+^ deficiency induces cell wall loosening to promote faster fiber elongation

Ca^2+^ is also a necessary component of cell wall pectin, and its deficiency blocks cell wall formation and induces tissue browning and necrosis (White and Broadley, 2003). In previous studies, Ca^2+^ deficiency in tomato has been shown to increase the activities of polygalacturonase and pectin methylesterases, resulting in excessive cell expansion (Corden, 1965). Tomato fruit appears to be more susceptible to blossom-end rot during the early phase of rapid cell expansion (Ho and White, 2005).

In this study, we found that Ca^2+^ deficiency induced ovule expansion and fiber elongation that was coupled with the specific up-regulation of *PSK*, *EXP*, and *XTH* (Fig. 6A and Supplementary Fig. S5), which encode cell wall loosening proteins that are important for elongation. Overexpression of *GhPSK* in cotton increases the endogenous level of PSK-α and promotes fiber cell elongation, resulting in longer and finer fibers that is associated with a reduced K^+^ efflux (Han *et al.*, 2014). Co-overexpression of GhEXPA1 (α-expansin protein) and GhRDL1 proteins in cotton plants resulted in up to 40% higher fiber yield per plant, but with no adverse effects on fiber quality and vegetative growth. However, overexpression of *GbEXPATR*, which encodes a truncated protein lacking the normal C-terminal polysaccharide-binding domain of α-expansin and is specifically expressed in *G. barbadense*, resulted in longer, finer and stronger fibers coupled with significantly thinner cell walls (Xu *et al.*, 2013; Li *et al.*, 2016). XTH activity was found to be higher in *Gb* fibers and the levels of its substrate xyloglucan in *Gb* fibers were lower than in *Gh* fibers (Avci *et al.*, 2013). Cotton plants overexpressing *GhXTH1* showed an increased XTH activity and produced mature fibers that were 15–20% longer than in the wild-type (Michailidis *et al.*, 2009; Lee *et al.*, 2010; Shao *et al.*, 2011).

Based on these above, we conclude that fiber elongation induced by Ca^2+^ deficiency might also be due to the up-regulation of cell wall loosening genes such as *PSK*, *EXP*, and *XTH.*

### Maintenance of optimal high levels of K^+^ is essential for Ca^2+^ deficiency-induced fiber elongation

Ca^2+^ deficiency induced an increase in K^+^ and in the K^+^/Ca^2+^ ratio in ovules, which contributed to longer fibers compared to normal Ca^2+^ conditions (Fig. 6). K^+^ has high mobility and preferentially accumulates in the cytoplasm and vacuole, and it contributes to the maintenance of osmotic pressure and cell expansion (Maathuis, 2009). Recently, it has reported that low K^+^ availability induced premature senescence and adversely affected cotton fiber properties, including fiber length, fiber strength, and lint weight (Yang *et al.*, 2016*a*, 2016*b*). K^+^ is also the major osmotic factor affecting fiber length, and absorptive capacities in the upper fruiting branches might affect fiber elongation and final length under low-K^+^ conditions (Yang *et al.*, 2016*a*). Additional quantitative analysis has revealed that K^+^ deficiency induces accelerated fiber cellulose accumulation and carbohydrate acquisition, which causes reduced fiber strength (Yang *et al.*, 2016*b*). During cotton fiber development, coincident with the transient closure of the plasmodesmata (10–16 DPA), the sucrose transporter *GhSUT1* and high-affinity K^+^ uptake gene *GhKT1* are preferentially expressed at 10 DPA, driving the rapid phase of elongation (Ruan *et al.*, 2001). The longer duration of plasmodesmatal closure at the base of the fiber in *Gb* compared to *Gh* is predicted to allow a high turgor to persist for longer in *Gb* fibers (Ruan *et al.*, 2004). After 15 DPA, the *Gh* fiber elongation rate begins to decrease, but in *Gb* fibers elongation is maintained to 20 DPA (Wang *et al.*, 2010). This was found to be associated with patterns of K^+^ homeostasis during fiber elongation, with the peak of accumulation occurring at 15 DPA in *Gb*, compared to 10 DPA in *Gh* (Fig. 1), suggesting that sufficient K^+^ in elongating fibers maintains a longer elongation stage.

### GhCIPK6 mediates the uptake of K^+^ in ovules and fibers under Ca^2+^-deficient conditions

Much evidence exists to show that the absorption of elements is controlled by an array of genes (Baxter, 2009; Zhu *et al.*, 2009). Under low-K^+^ conditions, roots of Arabidopsis use different strategies to adapt their growth, including the activation of ROS, Ca^2+^ signaling, and hormonal changes. ROS and Ca^2+^ signaling induce the up-regulation of *AtHAK5*, *AtCIPK6*, *AtCIPK23*, *AtCBL10*, *AtRHD2*, and *AtRCI3* genes or activate the assimilation of K^+^ by AtAKT1/2 or AtKUP3 transporters (Wang and Wu, 2013; Chérel *et al.*, 2014; Shin, 2014).

*CIPK6*, *RHD2*, *HAK5*, and *RCI3* genes were induced by low K^+^ availability, and *GhCIPK6* was induced by Ca^2+^ deficiency (Fig. 7 and Supplementary Table S5). The increased content of K^+^ and increased K^+^/Ca^2+^ ratio in Ca^2+^-deficient cultured ovules and fibers indicates that the K^+^ uptake system is activated by Ca^2+^ deficiency (Fig. 6). Reduced K^+^ and Ca^+^ deficiency might activate the same signaling pathways to increase K^+^ uptake through *GhCIPK6* (Fig. 7 and Supplementary Fig. S7). The increased sensitivity and reduced K^+^ content of *GhCIPK6*-silenced plants suggests that *GhCIPK6* is involved in the uptake of K^+^ (Fig. 7C, D and Supplementary Fig. S8). Ovules overexpressing *GhCIPK6* showed an increased sensitivity to Ca^2+^ deficiency under reduced K^+^ (K0) or sufficient K^+^, while *GhCIPK6*-silencing resulted in a higher tolerance to Ca^2+^ deficiency (Fig. 7E). These results suggest that GhCIPK6 mediates the uptake of K^+^ in ovules and fibers under Ca^2+^-deficient conditions. In Arabidopsis, the transcript of *AtCIPK6* increases upon K^+^ starvation and the CBL4-CIPK6 complex modifies AKT2, a *Shaker*-type of K^+^ channel, by increasing the translocation of AKT2 from the endoplasmic reticulum to the plasma membrane (Held *et al.*, 2011). This translocation of AKT2 is important for K^+^ circulation in the phloem and surrounding cells (Michard *et al.*, 2005; Latz *et al.*, 2007; Gajdanowicz *et al.*, 2011).

In conclusion, our study provides new evidence that mineral elements are necessary for fiber development, among which Ca^2+^ and K^+^ are the most important. We also provide evidence that crosstalk occurs between Ca^2+^ and K^+^, the kinase *GhCIPK6*, and the hormones ABA and JA to control cotton fiber development, illustrating the complexity of nutritional responses in plants.

## Supplementary Data

Supplementary data are available at *JXB* online.

Fig. S1. Illustration of the procedures for the ovule culture system.

Fig. S2. Ionome quantification of Fe, Zn, Mn, and B by ICP-MS in ovules and fibers at different development stages.

Fig. S3. Comparison of phenotypes and lengths of fibers harvested from cotton either grown in the field or in BT growth medium.

Fig. S4. Phenotypes and fiber lengths measured from treatments containing Fe, Zn, Mn, B, Mo, Co, and Cu at different concentrations in BT growth medium.

Fig. S5. Screening and analysis of differentially expressed genes in fibers cultured with three levels of K^+^ for 10 d.

Fig. S6. qRT-PCR verification of the Ca^2+^ deficiency-induced genes *PSK*, *EXP*, and *XTH* in ovules treated with different levels of Ca^2+^ or K^+^ for 5 d.

Fig. S7. Phylogenetic analysis of CIPK (CBL-interacting serine-threonine protein kinases) proteins and the response of GhCIPK6 to reduced K^+^ or Ca^2+^ deficiency.

Fig. S8. **A**nalysis of K^+^ content in the *GhCIPK6*-suppressed line (Ri16) and wild-type cotton under Ca^2+^ deficiency.

Fig. S9. Effect of the Ca^2+^ chelator EGTA on fiber development under different Ca^2+^ or K^+^ conditions after 3 d culture.

Table S1. List of primers used for qRT-PCR in this study.

Table S2. Fiber quality parameters of three cotton varieties.

Table S3. List of essential mineral element concentrations in the third leaf from the top of *G. hirsutum* TM-1.

Table S4. List of enriched GO terms for DEGs that were up-regulated in fibers in the K0 treatment compared with K1.

Table S5. List of ROS generation and scavenging genes expressed in response to reduced K^+^ or Ca^2+^ deficiency, based on the RPKM value from RNA-seq analysis.

Table S6. List of enriched GO terms for 1262 up-regulated genes in the Ca0K0.5 treatment compared with Ca1K0.5.

Table S7. List of enriched GO terms for 1132 up-regulated genes in the Ca0K0.5 treatment compared with Ca8K0.5.

Table S8. List of enriched GO terms for 774 genes up-regulated in the Ca0K0.5 treatment compared with both Ca1K0.5 or Ca8K0.5.

Table S9. K^+^ and Ca^2+^ concentrations determined by ICP-MS in Ca^2+^-deficient growth medium treated with the Ca^2+^ chelator EGTA for 3 d.

## Supplementary Material

Supplementary Figures S1-S9

Supplementary Tables S1-S8
