# Supplementary material for: Interaction between calcium and potassium modulates elongation rate in cotton fiber cells
Source: J Exp Bot. 2017 Oct 13;68(18):5161–75. doi: 10.1093/jxb/erx346 (PMC5853336; doi:10.1093/jxb/erx346)
Supplement: Supplementary Figures S1-S9 [file erx346_suppl_supplementary_figures_s1_s9.pdf]

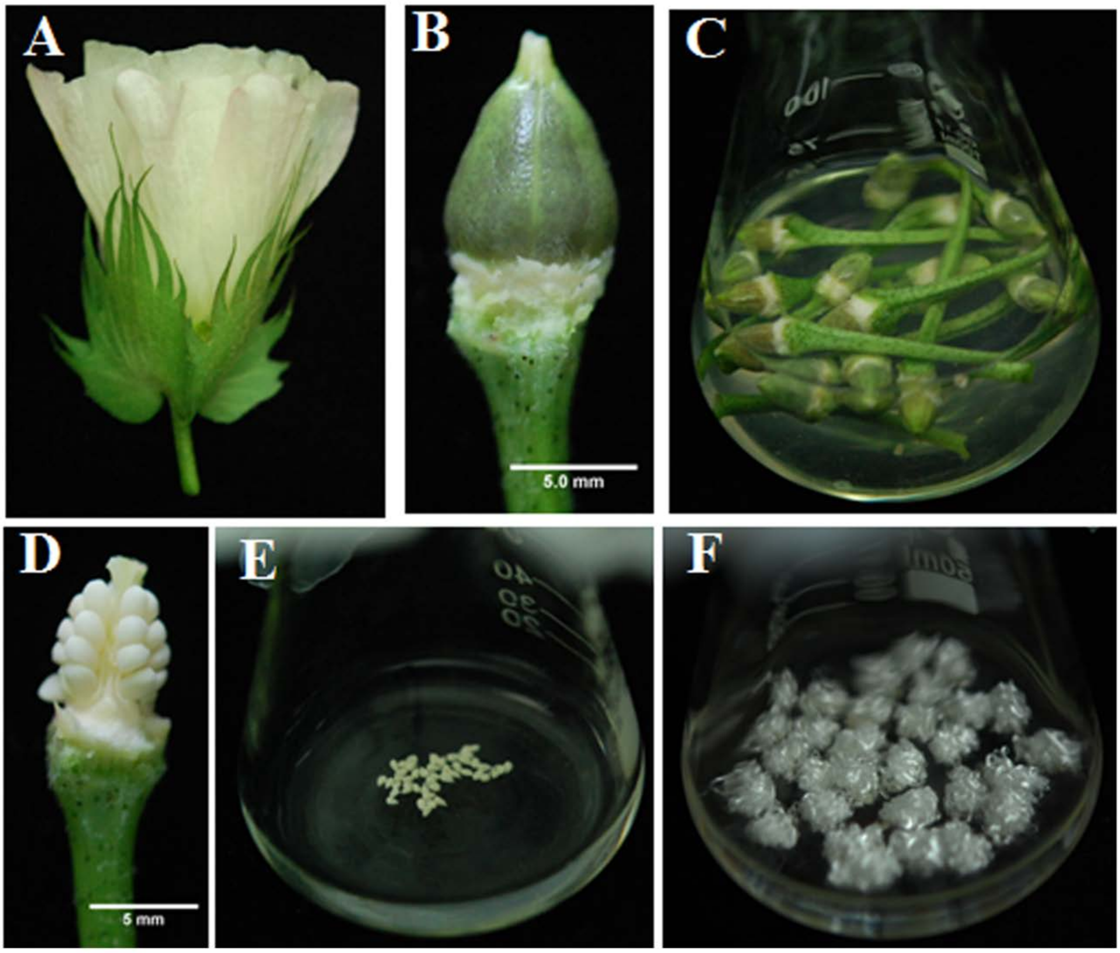

Supplementary Fig. S1 Procedures of the ovule culture system. (A) Cotton flower on the day of anthesis. (B) Ovary with sepal, petal, stamens and pistil removed. (C) Ovaries are sterilized with 0.1% (w/v) $HgCl_2$. (D) Ovary with shuck removed. (E) Intact ovules were floated on liquid medium. (F) Ovules covered with fibers after 8 days culture at 30℃ in the dark.

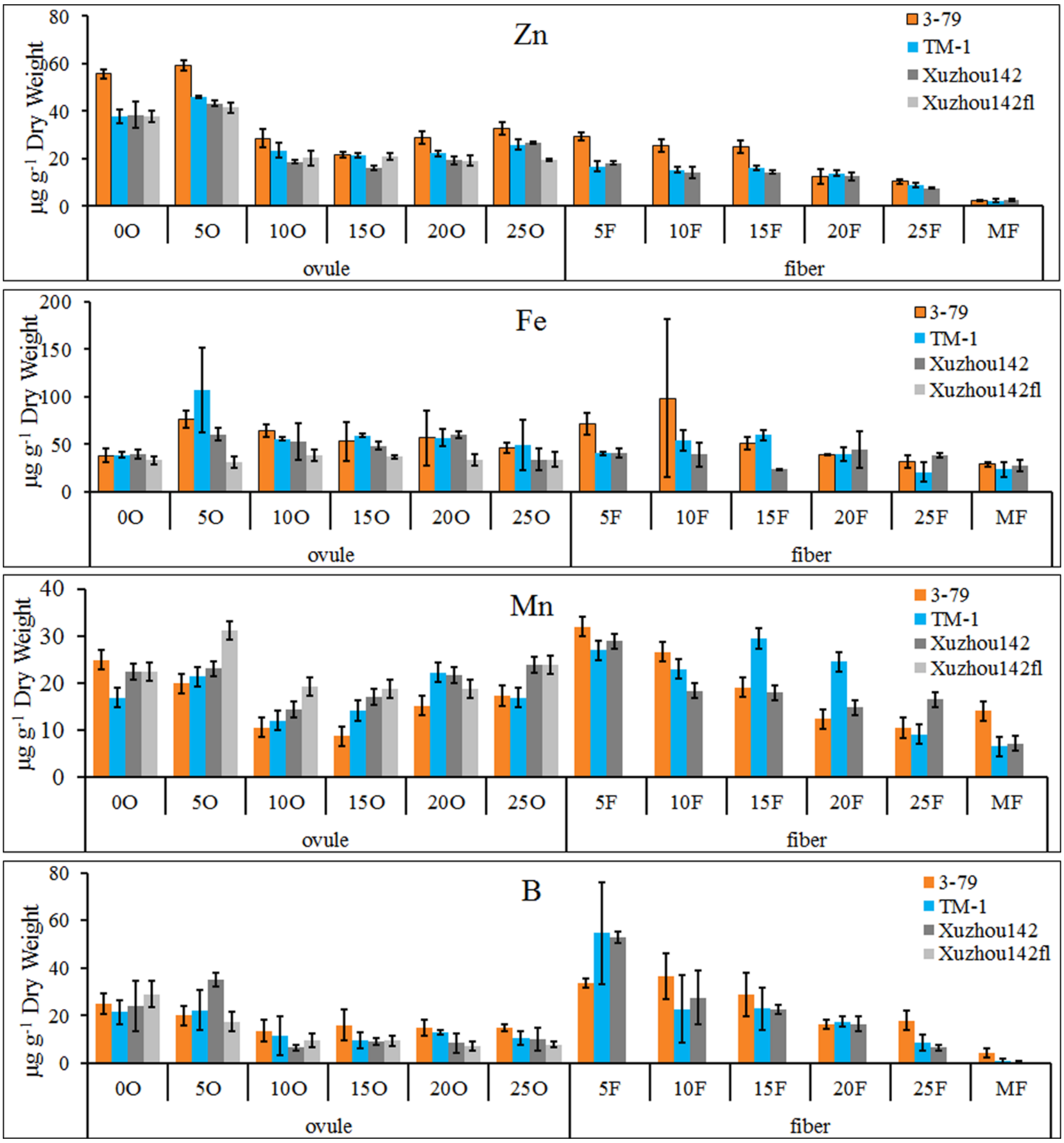

Supplementary Fig. S2 Ionome quantification of Fe (iron), Zn (zinc), Mn (manganese) and B (boron) by ICP-MS (inductively coupled plasma mass spectrometry) in ovules and fibers at different development stages. *G. barbadense* 3-79, *G.hirsutum* TM-1, Xuzhou 142 and *xu142-fl*. Mean ± sd, n=3. 0O-25O, 0 DPA to 25 DPA ovules; 5F-25F, 5 DPA to 25DPA fibers; MF, mature fiber.

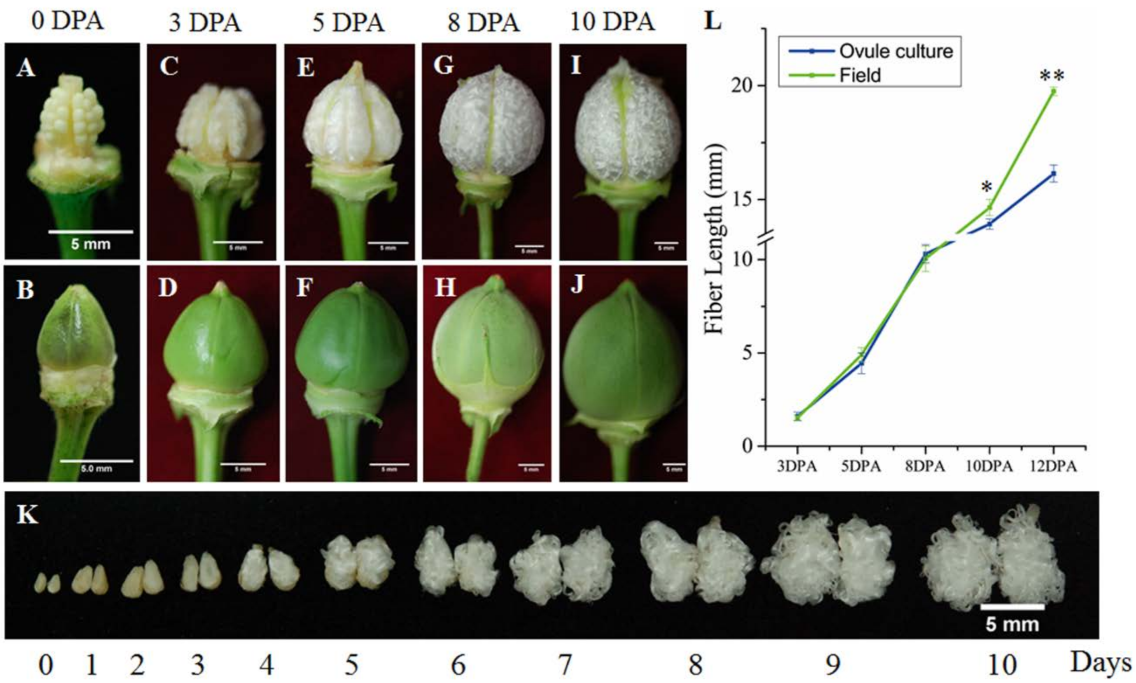

Supplementary Fig. S3 Comparison of phenotypes and lengths of fibers harvested from field and BT medium. Bars = 5 mm. A and B, the 0 DPA (days post anthesis) ovary harvested from field. C and D, 3 DPA cotton boll harvested from field. E and F, 5 DPA cotton boll harvested from field. G and H, 8 DPA cotton boll harvested from field. I and J, 10 DPA cotton boll harvested from field. K, Dynamic shape of fiber-bearing ovules after culture in BT medium from 0 day to 10 days. L, Dynamic length trends of fiber harvested from field and BT medium. Mean ± sd, n=3. Asterisk above the bars indicated that the difference is significant at * $P < 0.05$, or ** $P < 0.01$ (Student's t test).

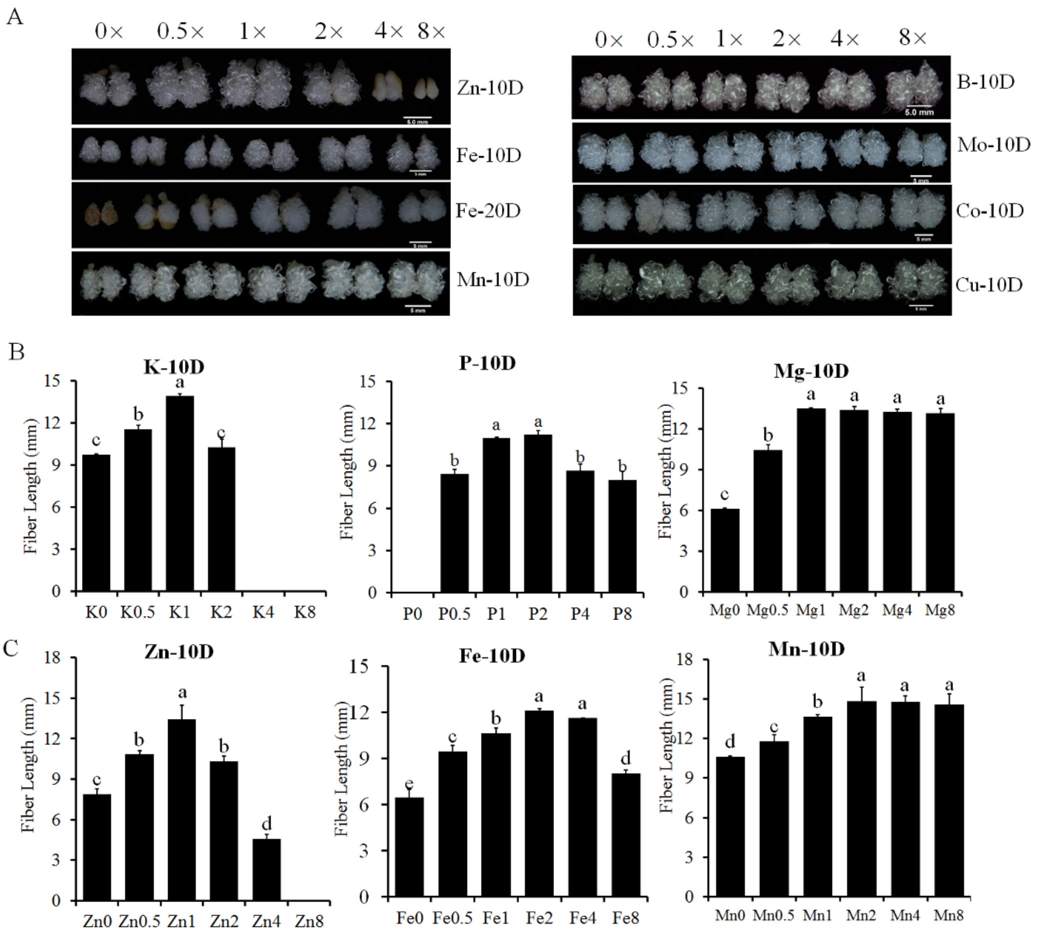

Supplementary Fig. S4 Phenotypes and fiber lengths measured from treatments containing mineral elements (Fe, Zn, Mn, B, Mo, Co and Cu) at different concentrations (0, 0.5-, 1-, 2-, 4- and 8-fold levels) in BT medium. (A) Phenotype of ovule and fiber treated with different levels of micro-elements. Number 1 indicates the optimum concentration in the ovule culture medium, a 0 indicates the medium for ovule cultured without the element, and 0.5, 2, 4 and 8 indicate element concentration in medium were 0.5-, 2-, 4- and 8-fold of the standard concentration, respectively. 10D or 20D indicates the ovules were cultured for 10 days or 20 days. (B) Length of fiber cultured for 10 days with different treatments. Mean ± sd, n=3. Different letters (a-f) above columns indicate that there is a significant difference at $P < 0.05$ (one-way ANOVA and Duncan's multiple comparisons).

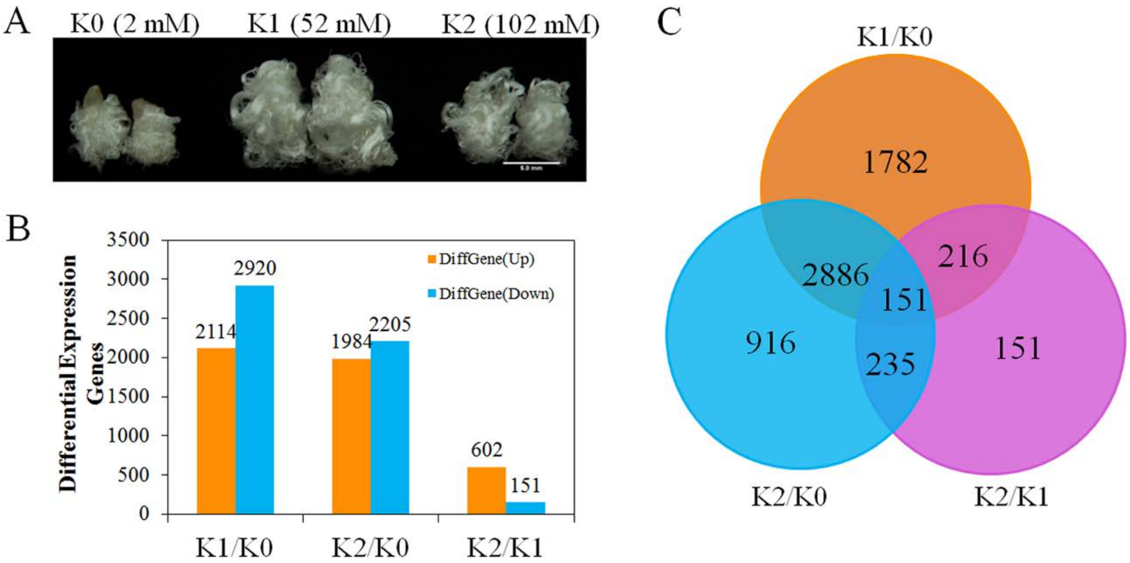

Supplementary Fig. S5 Screening and analysis of differentially expressed genes (DEGs) in fibers cultured with three levels of K^+ for 10 days. K0, 2 mM K^+; K1, 52 mM K^+; K2, 102 mM K^+. (A) Phenotype of fiber-bearing ovules cultured with three levels of K^+ in medium for 10 days. Bar=5 mm. (B) DEGs in 10 days fibers cultured with three levels of K^+. (C) Venn diagram analysis of DEGs responding to three levels of K^+.

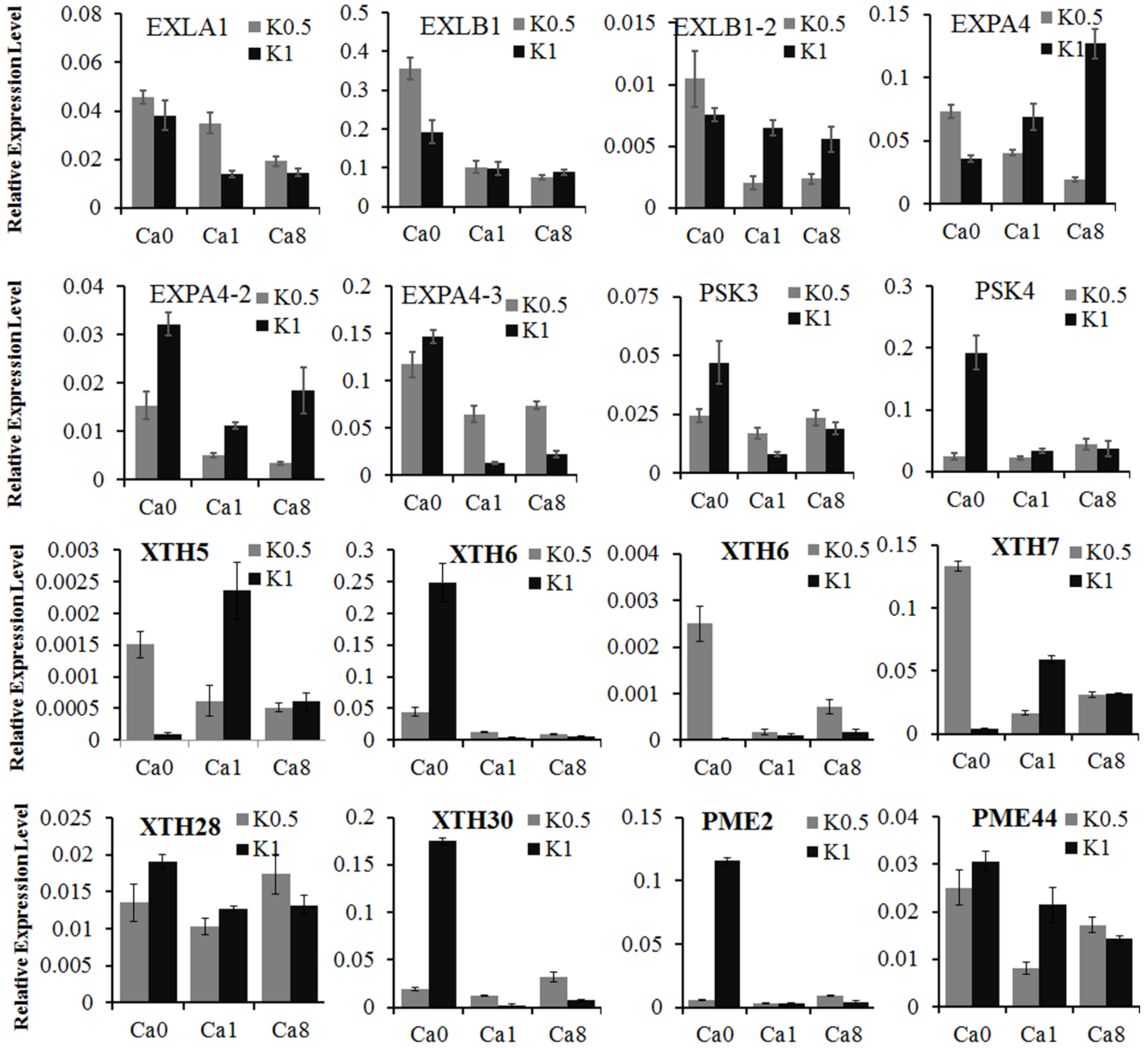

Supplementary Fig. S6 qRT-PCR verification of Ca^{2+} deficiency-induced genes *PSK*, *EXP* and *XTH* in ovules treated with different levels of Ca^{2+} or K^{+} for 5 days. Bars represent SD of three technological repeats. *GhUB7* was used as the internal control to normalize gene expression levels. K0.5, 27 mM K^{+}; K1, 52 mM K^{+}; Ca0, Ca^{2+} deficiency; Ca1, 3 mM Ca^{2+}; Ca8, 24 mM Ca^{2+}.

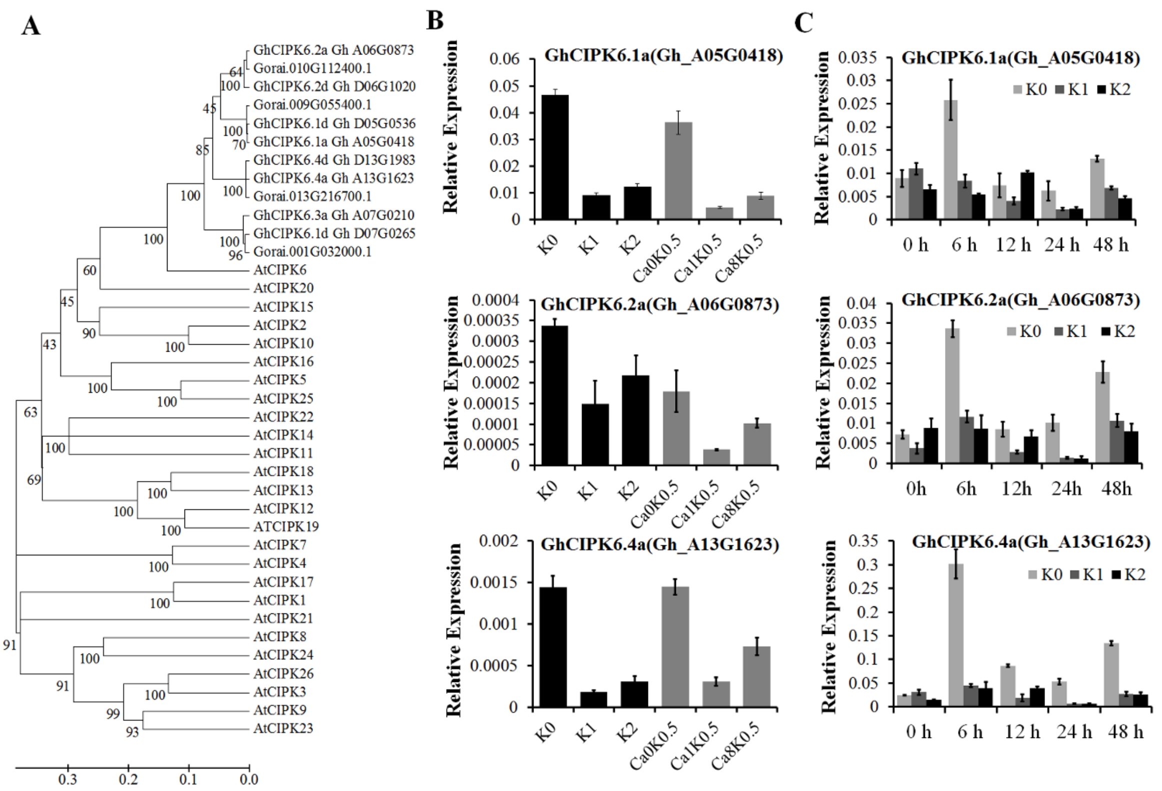

Supplementary Fig. S7 Phylogenetic analysis of CIPK (CBL-interacting serine-threonine protein kinases) proteins and the response of GhCIPK6 to the reduced K^+ or Ca^{2+} deficiency. (A) CIPK protein sequences from *G. raimondii* (*Gorai*), *G. hirsutum* (*Gh*) and *Arabidopsis thaliana* (*At*). The phylogenetic tree was constructed with MEGA 5.1 using the neighbor-joining method with 2000 replications. Molecular distances were calculated using the parameter of *p*-distance with pairwise deletion. (B) Expression levels of three cotton *CIPK6* genes in fibers cultured with different levels of K^+ or Ca^{2+}. K0, 2 mM K^+; K0.5, 27 mM K^+; K1, 52 mM K^+; K2, 102 mM K^+; Ca0, Ca^{2+} deficiency; Ca1, 3 mM Ca^{2+}; Ca8, 24 mM Ca^{2+}. (C) Expression patterns of three cotton *CIPK6* genes in fiber cultured for different time with different concentrations of K^+. *GhUB7* was used as the internal control to normalize the gene expression levels. The bars represent SD of three technological repeats.

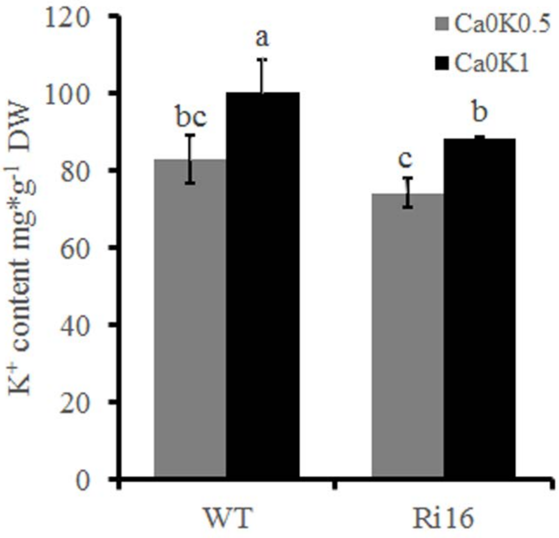

Supplementary Fig. S8 K^+ contents analysis in *GhCIPK6* suppressed line (Ri16) and wild type cotton under Ca^{2+} deficiency. K0.5, 27 mM K^+; K1, 52 mM K^+; Ca0, 0 mM Ca^{2+}.

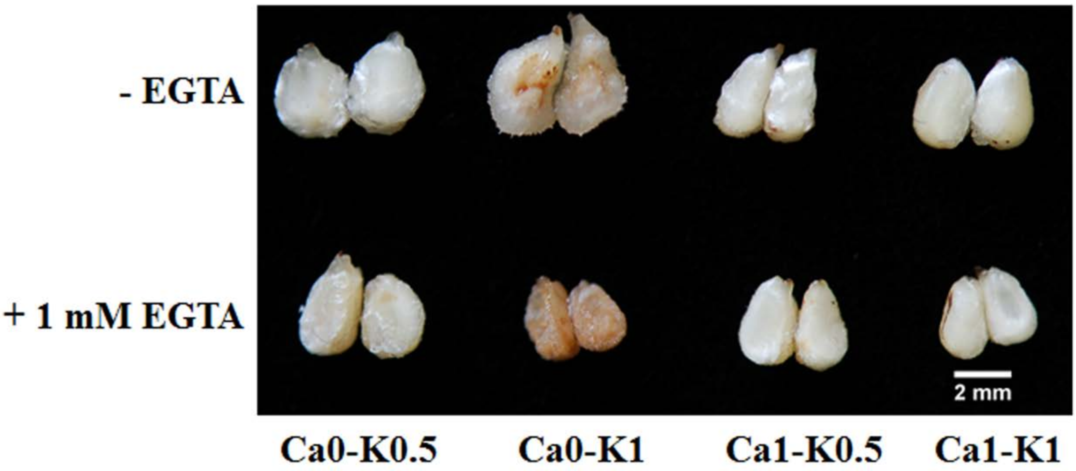

Supplementary Figure S9 Effect of Ca^{2+} chelator EGTA (Ethylene glycol-bis(2-aminoethylether) tetraacetic acid) on fiber development under different Ca^{2+} or K^+ conditions after 3 days culture.
